# Supplementary material for: Controlled human infection with Neisseria lactamica in late pregnancy to measure horizontal transmission and microbiome changes in mother–neonate pairs: a single-arm interventional pilot study protocol
Source: BMJ Open. 2022 May 17;12(5):e056081. doi: 10.1136/bmjopen-2021-056081 (PMC9119180; doi:10.1136/bmjopen-2021-056081)
Supplement: Author's manuscript [file bmjopen-2021-056081.draft_revisions.pdf]

BMJ Open

BMJ Open is committed to open peer review. As part of this commitment we make the peer review history of every article we publish publicly available.

When an article is published we post the peer reviewers' comments and the authors' responses online. We also post the versions of the paper that were used during peer review. These are the versions that the peer review comments apply to.

The versions of the paper that follow are the versions that were submitted during the peer review process. They are not the versions of record or the final published versions. They should not be cited or distributed as the published version of this manuscript.

BMJ Open is an open access journal and the full, final, typeset and author-corrected version of record of the manuscript is available on our site with no access controls, subscription charges or pay-per-view fees (http://bmjopen.bmj.com).

If you have any questions on BMJ Open's open peer review process please email info.bmjopen@bmj.com

BMJ Open

Controlled human infection with Neisseria lactamica in late pregnancy to measure horizontal transmission and microbiome changes in mother-neonate pairs: a single-arm interventional pilot study protocol

Journal:	*BMJ Open*
Manuscript ID	bmjopen-2021-056081
Article Type:	Protocol
Date Submitted by the Author:	03-Aug-2021

Complete List of Authors:	Theodosiou, Anastasia; University of Southampton, Clinical and Experimental Sciences<br>Laver, Jay; University of Southampton<br>Dorey, Robert; University of Southampton<br>Cleary, David; University of Southampton<br>Jones, Christine; University of Southampton<br>Read, Robert; University of Southampton

Keywords:	Paediatric infectious disease & immunisation < PAEDIATRICS, MICROBIOLOGY, Respiratory infections < THORACIC MEDICINE

SCHOLARONE™
Manuscripts

Full title: Controlled human infection with *Neisseria lactamica* in late pregnancy to measure horizontal transmission and microbiome changes in mother-neonate pairs: a single-arm interventional pilot study protocol

Running title: Protocol for *N. lactamica* controlled human infection in pregnancy

Authors

Anastasia A Theodosiou[1], Jay R Laver[1], Robert B Dorey[1], David W Cleary[1], Christine E Jones[1], Robert C Read[1]

Author affiliations

[1]University Hospital Southampton, Tremona Road, SO166YD Southampton

Corresponding author

Dr. Anastasia A Theodosiou

at1u17@soton.ac.uk

00447748479418

LC72, C Level South Academic Block, University Hospital Southampton, Tremona Road, SO166YD Southampton

For peer review only

Abstract

Introduction: Infant upper respiratory microbiota are derived partly from the maternal respiratory tract, and certain microbiota are associated with altered risk of infections and respiratory disease. *Neisseria lactamica* is a common pharyngeal commensal in young children, and is associated with reduced carriage and invasive disease by *Neisseria meningitidis*. Nasal inoculation with *N. lactamica* safely and reproducibly reduces *N. meningitidis* colonisation in healthy adults. We propose nasal inoculation of pregnant women with *N. lactamica*, to establish if neonatal pharyngeal colonisation occurs after birth, and to characterise microbiome evolution in mother-infant pairs over one month post-partum.

Methods and analysis: 20 healthy pregnant women will receive nasal inoculation with *N. lactamica* (wild type strain Y92-1009) at 36-38 weeks gestation. Upper respiratory samples, as well as optional breastmilk, umbilical cord blood and infant venous blood samples, will be collected from mother-infant pairs over one month post-partum. We will assess safety, *N. lactamica* colonisation (by targeted polymerase chain reaction) and longitudinal microevolution (by whole genome sequencing), and microbiome evolution (by 16S rRNA gene sequencing).

Ethics and dissemination: This study has been approved by the London Central Research Ethics Committee (21/PR/0373). Findings will be published in peer-reviewed open-access journals as soon as possible.

Trial registration number (ClinicalTrials.gov): NCT04784845

For peer review only

Keywords

Controlled human infection; *Neisseria lactamica*; microbiome; respiratory; neonatal; pregnancy

Article summary: Strengths and limitations of this study

1. To our knowledge, this will be the first study to investigate horizontal respiratory commensal acquisition in neonates following controlled human infection in pregnancy.
2. Controlled infection of adults with *Neisseria lactamica* is well-characterised, with data regarding safety, optimum dose, immune responses and genomic microevolution from over 400 adult participants to date.
3. Analysis of paired mother-infant upper respiratory microbiome samples will enrich our understanding of neonatal microbiome acquisition and evolution, and may identify other candidate commensals for future inoculation studies.
4. A limitation of this study is that the small number of participants may be insufficient to demonstrate a significant association between *N. lactamica* inoculation and observed microbiome changes or, in particular, a protective effect against *N. meningitidis* colonisation.
5. This proof-of-concept study will provide the preliminary data needed to plan future research, such as immunological analyses, or even direct neonatal inoculation with *N. lactamica*.

For peer review only

Acronyms / abbreviations used

- CFU – colony-forming units
- GM – genetically modified
- GMP – Good manufacturing practice
- HRA – Health Research Authority
- NHS – National Health Service
- PCR – polymerase chain reaction
- REC – Research Ethics Committee
- rRNA – ribosomal ribonucleic acid
- RTI – respiratory tract infection
- URT – upper respiratory tract
- X-gal - 5-bromo-4-chloro-3-indolyl-β-D-galactopyranoside

Introduction

Upper respiratory pathobionts and infant disease

Upper respiratory tract (URT) pathobionts are common colonising bacteria capable of causing disease in immunocompetent individuals (e.g. *Neisseria meningitidis, Streptococcus pneumoniae, Haemophilus influenzae*). Although harmless in many hosts, these remain a leading cause of global childhood death and disease, especially in the first year of life: globally, 492,000 and 89,000 infants die each year due to respiratory tract infection (RTI) and meningitis, respectively[1]. While many infant infections are vaccine-preventable, global access to and acceptance of vaccines remains incomplete, such that 1.5 million vaccine-preventable childhood deaths occur each year[2]. Furthermore, existing vaccines do not protect against all important childhood respiratory pathogens (such as respiratory syncytial virus and some serotypes of *S. pneumoniae*), and vaccinations are not routinely given in the first two months post-partum, when risk of invasive infection is highest. Rising global antimicrobial resistance compounds these challenges, with 214,000 neonates dying of resistant infections each year[3].

For peer review only - http://bmjopen.bmj.com/site/about/guidelines.xhtml

Recent research has highlighted the relationship between URT pathobionts, childhood disease, and the evolving infant microbiome (the site-specific total microbial community)[4]. Taxonomic profiling (e.g. by bacterial 16S rRNA gene sequencing) demonstrates distinct URT microbiota during acute RTI, and that URT microbiome perturbation is associated with RTI severity and recurrence, childhood chronic wheeze, and early allergic sensitisation[5-8].

Infant RTIs are often preceded by microbiome changes, including increased relative abundance of URT pathobionts and loss of topography (blurring of distinction between microbiomes at adjacent anatomical sites)[9], suggesting a possible causal role in developing infection. However, as up to 93% of infants are colonised with at least one URT pathobiont at any time[10], pathobiont colonisation alone is not necessarily associated with infection: rather, the presence of pathobionts may indicate a state of dysbiosis (microbial dysregulation), in which disease-causing bacteria and viruses can cause infection in a previously resilient ecological niche.

Longitudinal cohort microbiome studies suggest that infant URT flora are acquired at least in part from their mothers: there is greater overlap between a neonate's oral flora and that of its own mother than unrelated mothers, and shared bacterial strains (detected by whole genome sequencing) are more long-lived in an infant's mouth than non-maternal strains[11]. Moreover, a subset of maternal oral strains account for a disproportionately large share of their infants' oral flora.

Can upper respiratory microbiota be manipulated to prevent infant disease?

In the future, it is possible that simple methods of modifying the infant nasopharyngeal microbiome could provide useful adjunct protection in the early months of life[12]. Previously, neonatal nasal inoculation with low-virulence *Staphylococcus aureus* strain 502A has been shown to reduce colonisation by high-virulence *S. aureus* strain 80/81 in response to an outbreak[13], while pharyngeal inoculation with strain 215 alpha-haemolytic streptococci reduced pharyngeal

For peer review only

pathobionts (*Escherichia coli, Klebsiella pneumoniae, S. aureus*) in neonatal intensive care inpatients[14]. Furthermore, a variety of probiotics (living microorganisms administered to confer a health benefit) have been trialled in children and adults, with some reporting a reduction in upper RTI incidence and duration[15]. However, high quality efficacy and microbiome data is lacking, with significant inter-study variation in the bacterial strain (usually lactobacilli, streptococci and bifidobacteria), preparation (usually capsules or dairy supplement), dose and regimen. That being said, there is evidence that many probiotics are safe in pregnancy, neonates and lactating mothers, with some already available over-the-counter to these groups[16].

Controlled human infection with *Neisseria lactamica*

Unlike probiotic studies (which often employ proprietary food supplements with uncontrolled concentrations of bacteria), controlled human infection offers a robust model for studying the impact of a defined bacterial inoculum on a human host. Our research group has previously developed a safe and reliable controlled human infection model using nasal inoculation with 10^4-10^5 colony-forming units (CFU) *N. lactamica* strain Y92-1009 in healthy adults[17]. In randomised blinded placebo-controlled studies, we have characterised *N. lactamica* colonisation density, duration, cellular and humoral immune responses, and genomic microevolution[18], observing colonisation rates of up to 85% and no serious adverse reactions following over 400 inoculations.

N. lactamica is a common acapsular pharyngeal commensal that does not cause invasive disease in immunocompetent hosts. Colonisation peaks at over 40% in 1-2 year old children[19], before falling to less than 10% in adulthood[20], and *N. lactamica* carriage correlates inversely with *N. meningitidis* colonisation[21] and invasive disease[20]. Indeed, *N. lactamica* controlled human infection in healthy adults reduced naturally-acquired *N. meningitidis* carriage from 18% to 8% (both by displacing existing colonisation and preventing new acquisition), exceeding the typical impact of glycoconjugate ACWY vaccination[17]. This displacement effect is not specific to a particular serogroup, unlike available meningococcal vaccines.

For peer review only

Furthermore, natural colonisation with *N. lactamica* has been observed to correlate inversely with invasive meningococcal disease[20], although this protection against invasive disease has not yet been demonstrated with *N. lactamica* inoculation.

Rationale for this study

The aim of this study is to establish if nasal inoculation with *N. lactamica* in pregnancy results in neonatal colonisation after birth, and to characterise the impact on the developing neonatal URT microbiome. If successful, this will provide a novel model for inducing and studying true person-to-person commensal acquisition in neonates (unlike traditional controlled human infection models, which capture inoculation-induced colonisation). Looking ahead, this may provide a new strategy for reducing *N. meningitidis* colonisation and disease in neonates, and of modifying neonatal microbiome development with a view to improving health outcomes.

For peer review only

Methods and analysis

Study overview

In this prospective controlled human infection study, 20 healthy pregnant women will receive nasal inoculation with 10^5 CFU *N. lactamica* (wild type strain Y92-1009) at 36+0 to 37+6 weeks gestation. Upper respiratory samples and breastmilk will be collected from mother-infant pairs 1 day, 1 week and 1 month post-partum, as well as an umbilical cord blood sample at delivery and an infant venous blood sample at 1 month post-partum. Safety outcomes and relevant clinical data (including mode of delivery, infant feeding and use of antimicrobials) will be recorded. Samples will be analysed by microbiological culture, targeted polymerase chain reaction (PCR), and sequencing to determine *N. lactamica* Y92-1009 presence, colonisation density, and longitudinal microevolution, as well as 16S rRNA gene sequencing to characterise microbiome evolution in mother-neonate pairs.

Study objective and endpoints

This study aims to establish whether horizontal *N. lactamica* transfer occurs from experimentally-colonised mothers to their neonates following nasal *N. lactamica* inoculation in late pregnancy, and to characterise the safety profile, colonisation kinetics, and impact on microbiome development in mother-neonate pairs (Table 1).

N. lactamica inoculum

Lyophilised (freeze-dried) *N. lactamica* (LyoNlac; strain Y92-1009, sequence type 3493, clonal complex 613) will be produced and stored under Good Manufacturing Practice (GMP)-like standards at the University of Southampton. Lyophilised inoculum stocks can be stored at 4°C (unlike frozen stocks requiring storage at -80°C). LyoNlac will be reconstituted in phosphate buffered saline (PBS) and diluted to a concentration of 10^5 CFU/ml for inoculation. In our previous work,

this dose resulted in *N. lactamica* colonisation in 100% of 10 adults receiving lyophilised inoculum and 85% of 20 adults using frozen inoculum (i.e. 90% overall). Recovery of viable bacteria is nearly 100% immediately following lyophilisation, and remains over 10^7 CFU/ampoule if reconstituted 3 months after lyophilisation (unpublished data).

Recruitment of study volunteers

Healthy adult volunteers in the second and third trimesters of pregnancy will be approached using ethically-approved advertisements and direct contact by research midwives or a study doctor. Individuals who express an interest will be given a participant information sheet, and invited to attend a screening visit. Participants will be offered reimbursement for their time, travel and inconvenience.

Study visits and procedures

Screening

Interested individuals will attend a screening visit at 34+0 to 36+6 weeks gestation, to discuss the study timeline and procedures (Table 2), and assess eligibility (Table 3). Eligible individuals who provide informed consent at screening will be considered enrolled. A detailed medical history will be taken, followed by respiratory sampling to assess for baseline natural *N. lactamica* carriage.

Inoculation

Eligible consenting volunteers who are not already naturally colonised with *N. lactamica* will receive nasal inoculation with 10^5 CFU *N. lactamica* Y92-1009, using a sterile pipette will be used to deliver 0.5ml inoculum to each nostril. Enrolled volunteers who are already naturally colonised with *N. lactamica* will not be inoculated. However, they will be invited to remain in the study as an opportunistic comparison cohort, and will complete all post-partum visits and sampling as for the inoculated mother-neonate pairs (Table 2).

For peer review only

Post-partum follow-up

Mother-neonate pairs will be reviewed one day, one week and one month post-partum. At each visit, maternal consent for neonatal sampling will be re-affirmed, and respiratory samples will be collected (Table 2). An optional umbilical cord blood sample (Visit 3) and neonatal venous blood sample (Visit 5) will be collected, as well as relevant clinical data (including mode of delivery, infant feeding and use of antimicrobials). Optional breastmilk samples will also be collected at each post-partum visit (up to 5ml each at Visits 3 to 5). Volunteers may decline optional blood and breastmilk sampling without affecting their enrolment in or completion of the rest of the study.

Volunteers' experiences of participation (including acceptability and tolerability of inoculation and maternal and infant sampling) will be captured using optional questionnaires, to help us identify areas for improving volunteer experience in current and future studies.

Sample processing

N. lactamica *identification*

Working in a class 2 microbiological safety cabinet in a Containment Level 2 laboratory, all respiratory samples will be suspended in 1ml storage medium (10% glycerol in 0.1% diethylpyrocarbonate [DEPC]-treated water). Agar selective for *N. lactamica* (GC agar supplemented with 5-bromo-4-chloro-3-indolyl-β-galactopyranoside [X-gal]) will be inoculated with sample solution, and all remaining sample solution will be split into 200μl aliquots for storage in a secure, temperature-monitored -80°C freezer. Following 24-48 hours incubation, colonies with typical *N. lactamica* morphology (blue on GC X-gal agar) will undergo identification by oxidase reaction, Gram stain, microscopy, and analytical profile index (API-NH, bioMérieux, France), with the option of matrix-assisted laser desorption/ionization time-of-flight (MALDI-TOF) if API-NH is inconclusive. *N. lactamica* density will be quantified and isolates will be stored at -80°C, and Y92-1009 strain identity will be confirmed using targeted PCR.

For peer review only - http://bmjopen.bmj.com/site/about/guidelines.xhtml

Breastmilk will also be plated onto selective agar to examine for evidence of *N. lactamica*, and residual samples will be stored at -80°C for microbiome analysis.

Microbiome analysis

Paired mother-infant frozen sample aliquots will be transported to the University of Edinburgh Centre for Inflammation Research for 16S rRNA gene sequencing[6]. Resulting sequence reads will undergo taxonomic classification and statistical analysis to compare mothers and infants, and (assuming some neonates become colonised with *N. lactamica*) colonised and uncolonised infants, across different sample types and study time points.

N. lactamica *sequencing*

If any neonates become colonised with *N. lactamica*, paired mother-infant *N. lactamica* isolates will undergo whole genome sequencing to assess for differences in *N. lactamica* microevolution in mother-infant pairs. Resulting sequences will be mapped to a closed *N. lactamica* Y92-1009 reference genome for comparison.

Sample size

To our knowledge, this proof-of-concept represents the first respiratory controlled human infection study in pregnancy. As such, it is not possible to accurately define the sample size needed to conclusively rule out induced horizontal transfer of *N. lactamica*. A pragmatic sample size of 20 inoculated volunteers has been selected: based on our earlier work, *N. lactamica* colonisation is expected in 18 (90%) of volunteers inoculated with 10^5 CFU *N. lactamica*. This sample would therefore fail to detect horizontal transfer only if it occurs in less than 1 in 18 infants (<6%), in which case it would be impractical and even unethical to pursue horizontal *N. lactamica* controlled human infection as a method of studying and manipulating infant commensalisation. By one month of age, 2.3% of infants naturally carry *N. lactamica*[17]. Based on a plot of lower confidence limits against observed infant *N. lactamica* colonisation for a sample of

18 experimentally-colonised volunteers, infant *N. lactamica* colonisation greater than 22% (95% CI [2.3%, 41.7%]) would demonstrate that inoculation-induced carriage exceeds natural *N. lactamica* carriage (Figure 1). Although we do not expect lower colonisation efficiency in pregnant adults, this sample is sufficient to detect significantly lower colonisation in pregnant compared with non-pregnant adults if 13 (65%) or fewer women become colonised with *N. lactamica* following inoculation (n=16 needed for power=0.8, α=0.05).

We expect to screen 24 volunteers to reach our target of 20 inoculations, allowing for 10% dropout and 10% baseline natural *N. lactamica* carriage[18]. It is not clear how maternal and/or neonatal antibiotics may influence *N. lactamica* carriage and microbiota. At the Principal Investigator's discretion, an additional volunteer may be enrolled and inoculated for every volunteer (or neonate) who receives antibiotics after inoculation, up to a maximum of 10 additional volunteers.

Patient and public involvement

During early study design, an interview-based Patient and Public Involvement (PPI) project was conducted with 12 pregnant women (unpublished data). All 12 women expressed approval that the proposed study should go ahead, 11 of whom would personally have liked to participate. These discussions led to changes in study design (such as offering follow-up visits in the volunteer's own home, and obtaining an umbilical cord rather than an infant venous blood sample at birth). During participant recruitment following study completion, the University of Southampton Public Engagement Research Unit will assist in showcasing the study at public engagement events.

For peer review only

Ethics and dissemination

Ethics and safety

Volunteer safety and ethical study conduct is of paramount importance. This study has been approved by the NHS Research Ethics committee (REC) and the Health Research Authority (HRA), and is sponsored by the University of Southampton. An External Safety Committee has been appointed to provide independent safety oversight. The *N. lactamica* Y92-1009 inoculum safety profile is well-characterised: over 400 adults have previously been inoculated with this *N. lactamica* strain (of whom 30 received lyophilised inoculum, and 54 received 10^5 CFU), with no serious adverse reactions reported to date[15] (unpublished data). Due to the use of yeast-based and soya-based products in inoculum production, individuals reporting allergies to yeast or soya will not be eligible for enrolment. Inoculation will take place in a clinical area with access to adult, obstetric and paediatric emergency services. Volunteers will be reviewed by a study doctor immediately prior to and 30 minutes after inoculation. Adverse events will be recorded throughout the study, and volunteers will be encouraged to report any concerns (including by telephone to the study doctor out-of-hours).

Mitigation strategies are in place to minimise the risk of SARS-CoV2 transmission to participants and study staff. Where possible, study visits will be conducted in the volunteer's home, practicing social distancing and appropriate personal protective equipment for non-aerosol generating procedures. If participants report symptoms of or contact with anyone with suspected or confirmed SARS-CoV2 infection, study visits will be rescheduled until testing or self-isolation as necessary.

Dissemination plan

Findings will be published in peer-reviewed journals as soon as is practicable. Participants will be provided with a lay summary of the study results once available.

Potential benefits and study impact

To our knowledge, this is the first respiratory controlled human infection study in pregnancy, and, if successful, would be the first to demonstrate induced person-to-person commensal transmission. This novel model could be used to characterise microbiome and immunological changes associated with induced commensalisation. Inducing *N. lactamica* colonisation in neonates may also provide a new strategy for reducing *N. meningitidis* colonisation and even disease in infants. Although many neonates are protected by trans-placental maternal antibodies, invasive meningococcal disease does rarely occur in infants, especially before vaccination[22]. Looking ahead, improved understanding of microbiome evolution following controlled human infection is necessary before inoculation in neonates (rather than in pregnant adults) can be considered.

Our team have recently trialled nasal inoculation in healthy non-pregnant adults using genetically-modified (GM) *N. lactamica* that expresses the meningococcal antigen NadA[23]. This GM inoculum has proved safe and effective at eliciting immune responses to NadA, providing a proof-of-concept that a harmless commensal may be used as a vehicle for prolonged mucosal exposure to an antigen of interest. Looking ahead, GM *N. lactamica* expressing antigens from other clinically-important paediatric respiratory pathogens could be explored, to complement existing childhood vaccination.

Conclusion

This protocol outlines a novel model for inducing and studying neonatal URT commensal acquisition and associated microbiome evolution. This model will in future be utilised to study neonatal mucosal immune responses to commensalisation, vaccination and infection, and to develop novel strategies of preventing infection in infancy.

For peer review only

Tables

Primary endpoint	Secondary endpoints
1. Confirmation of neonatal *N. lactamica* colonisation by selective culture of nasopharyngeal and saliva samples.	1. Confirmation of neonatal *N. lactamica* Y92-1009 colonisation by targeted PCR.<br>2. *N. lactamica* colonisation density in inoculated volunteers compared with their infants across study time points and sample types.<br>3. Microbiome composition across study time points and sample types in inoculated volunteers compared with their infants, and in colonised compared with uncolonised infants.<br>4. *N. lactamica* genome sequence for isolates derived from inoculated volunteers compared with their infants across study time points and sample types.
**Safety endpoints**<br>1. Percentage of inoculated volunteers with adverse reactions or serious adverse events within the study period.<br>2. Percentage of neonates with serious adverse events within the study period.	

Table 1. Primary, secondary and safety study endpoints. PCR: polymerase chain reaction.

	Screening (Visit 1)	Inoculation (Visit 2)	Birth (Visit 3)	Follow-up (Visits 4 and 5)	
Timeline	34+0 to 36+6 weeks gestation	36+0 to 37+6 weeks gestation	0-24 hours post-partum	7 +/- 3 days post-partum	28 +/- 3 days post-partum
Participant Information Sheet and consent form	+				
Clinical review	+	+	+	+	+
Nasal inoculation with *N. lactamica*		+			
Nasopharyngeal swabs (maternal)	+	+	+	+	+

For peer review only

Oropharyngeal swabs (maternal)	+	+	+	+	+
Saliva swabs (maternal)	+	+	+	+	+
Nasopharyngeal swabs (infant)			+	+	+
Saliva swabs (infant)			+	+	+
Umbilical cord blood			Optional		
Infant venous blood (up to 2ml)					Optional
Breastmilk (up to 5ml)			Optional	Optional	Optional
Visit location	NIHR-CRF or volunteer's own home	NIHR-CRF only	NIHR-CRF, inpatient area, or volunteer's own home	NIHR-CRF or volunteer's own home	NIHR-CRF or volunteer's own home

Table 2: Study visits and activities. TOPS: The Over-volunteering Prevention System; NIHR-CRF: National Institute for Health Research Clinical Research Facility, University Hospital Southampton.

Inclusion criteria (all must apply):

- Healthy adult aged 18 years or over on the day of enrolment.
- Singleton pregnancy, 34+0 to 36+6 weeks gestation on the day of enrolment.
- Documentation of a 20-week ultrasound scan with no life-limiting congenital anomalies, and no maxillofacial, otorhinolaryngolocial or neuroanatomical anomalies.
- Able and willing (in the Investigator's opinion) to comply with all study requirements.

For peer review only

▪ Able and willing to give written informed consent to participate in the study.<br>▪ Booked to receive antenatal care at University Hospital Southampton NHS Foundation Trust (including any associated community antenatal care facilities).
**Exclusion criteria (none must apply):**<br>▪ Any confirmed or suspected immunosuppressive or immunocompromised state, including: HIV infection; asplenia; recurrent severe infections; or use of immunosuppressant medication (for more than 14 days within the past 6 months, excluding topical and inhaled steroids).<br>▪ Planned use of immunosuppressant medication in later pregnancy or post-partum.<br>▪ Occupational, household or intimate contact with any immunosuppressed persons.<br>▪ Participation within the last 12 weeks in a clinical trial involving receipt of an investigational product, or planned use of an investigational product during the study period.<br>▪ Prior participation at any time in research studies involving inoculation with *N. lactamica.*<br>▪ Use of oral or intravenous antibiotics within 30 days prior to the inoculation visit.<br>▪ Planned use of oral or intravenous antibiotics at any time during the study period (e.g. for planned elective caesarean section or group B streptococcus colonisation).<br>▪ Allergy to soya or yeast.<br>▪ Previous stillbirth or neonatal death.<br>▪ Pre-pregnancy diabetes mellitus.<br>▪ Any other finding that may (in the Investigator's opinion): increase the risk to the volunteer (or their fetus/infant or close contacts) of participating in the study; affect the volunteer's ability to participate in the study and complete follow-up; or impair interpretation of study data.

For peer review only

Table 3. Inclusion and exclusion criteria. NHS: National Health Service; HIV: human immunodeficiency virus.

For peer review only

References

1. Roser M et al. Child and Infant Mortality. OurWorldInData.org Nov 2019. Retrieved Dec 2020 from: https://ourworldindata.org/child-mortality.
2. Gostin L et al. The public health crisis of underimmunisation: a global plan of action. Lancet Infect Dis 2020;20(1):e11-16.
3. Laxminarayan R et al. Access to effective antimicrobials: a worldwide challenge. Lancet 2016;387(10014):168-75.
4. Theodosiou AA et al. The infant pharyngeal microbiomes: origin, impact and manipulation. Curr Opin Infect Dis 2020;33(6):548-555.
5. Teo S et al. Dynamics of airway microbiota identify a critical window for interplay of pathogenic bacteria and allergic sensitization in childhood respiratory disease. Cell Host Microbe 2018;24(3):341–352.
6. Luna P et al. The association between anterior nares and nasopharyngeal microbiota in infants hospitalized for bronchiolitis. Microbiome 2018; 6(1):2.
7. Bosch A et al. Maturation of the infant respiratory microbiota, environmental drivers, and health consequences.Am J Respir Crit Care Med 2017;196(12):1582–1590.
8. Thorsen J et al. Infant airway microbiota and topical immune perturbations in the origins of childhood asthma. Nat Commun 2019;10(1):5001.
9. Man W et al. Loss of microbial topography between oral and nasopharyngeal microbiota and development of respiratory infections early in life. Am J Respir Crit Care Med 2019;200(6):760–770.
10. de Steenhuijsen Piters W et al. The role of the local microbial ecosystem in respiratory health and disease. Philos Trans R Soc B Biol Sci 2015;370(1675):20140294.
11. Ferretti P et al. Mother-to-infant microbial transmission from different body sites shapes the developing infant gut microbiome. Cell Host Microbe 2018;24(1):133–145.
12. Theodosiou AA et al. Manipulating the infant respiratory microbiomes to improve clinical outcomes: A review of the literature. J Infect 2021;82(6):247-252.
13. Shinefield H et al. Bacterial interference: its effect on nursery-acquired infection with Staphylococcus aureus. V. An analysis and interpretation. Am J Dis Child 1963;105:683–8.
14. Sprunt K et al. Abnormal colonization of neonates in an ICU: conversion to normal colonization by pharyngeal implantation of alpha haemolytic Streptococcus strain 215. Pediatr Res 1980;14(4):308–313.
15. Hao Q et al. Probiotics for preventing acute upper respiratory tract infections. Cochrane Database Syst Rev 2015;(2):CD006895.
16. Elias J et al. Are probiotics safe for use during pregnancy & lactation? Can Fam Physician 2011;57(3):299-301.
17. Deasy A et al. Nasal Inoculation of the Commensal Neisseria lactamica Inhibits Carriage of Neisseria meningitidis by Young Adults. Clin Infect Dis 2015;60(10):1512-20.
18. Pandey A et al. Microevolution of Neisseria lactamica during nasopharyngeal colonisation induced by controlled human infection. Nat Commun 2018;9(1):4753.
19. Bennett J et al. Genetic diversity and carriage dynamics of Neisseria lactamica in infants. Infect Immun 2005;73(4):2424.
20. Olsen S et al. Pharyngeal carriage of N meningitidis and N lactamica in households with infants within areas with high and low incidences of meningococcal disease. Epidemiol Infect 1991;106(3):445-57.

For peer review only

21. Gold R et al. Carriage of N meningitidis and N lactamica in infants and children. J Infect Dis 1978;137(2):112.
22. Kiray Bas et al. Neonatal infection with Neisseria meningitidis: analysis of a 97-year period plus case study. J Clin Microbiol 2014;52(9):3478-82.
23. Laver JR et al. A recombinant commensal bacteria elicits heterologous antigen-specific immune responses during pharyngeal carriage. Sci Transl Med. 2021;13(601).

Author contributions

All the authors contributed to the study design and submission for ethical approval. The first draft of this manuscript was prepared by Dr A Theodosiou, and edited and approved by all authors.

Funding statement

This work is supported by a Medical Research Council Clinical Research Training Fellowship (grant number MR/V002015/1) awarded to Dr Anastasia A Theodosiou. Robert B Dorey is a National Institute for Health Research (NIHR) Academic Clinical Fellow. Robert C Read is an NIHR Senior Investigator.

Competing interests statement

The authors have no competing interests to declare.

Word count

<4000 words

For peer review only

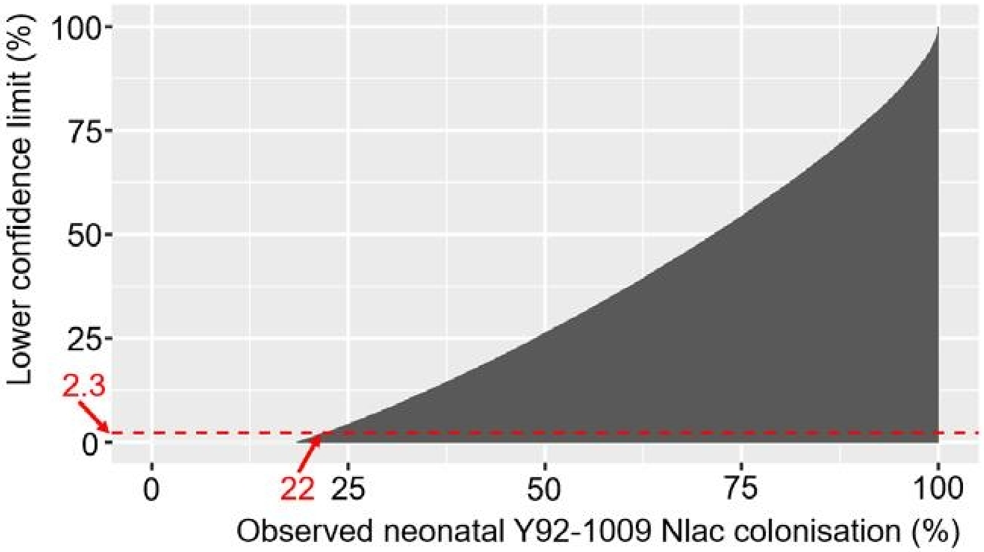

Figure 1: Lower confidence limit plot for inoculation-induced N. lactamica (Nlac) colonisation.

116x66mm (144 x 144 DPI)

BMJ Open

Controlled human infection with Neisseria lactamica in late pregnancy to measure horizontal transmission and microbiome changes in mother-neonate pairs: a single-arm interventional pilot study protocol

Journal:	*BMJ Open*
Manuscript ID	bmjopen-2021-056081.R1
Article Type:	Protocol
Date Submitted by the Author:	04-Mar-2022
Complete List of Authors:	Theodosiou, Anastasia; University of Southampton, Clinical and Experimental Sciences<br>Laver, Jay; University of Southampton<br>Dale, Adam; University of Southampton, Clinical and Experimental Sciences<br>Cleary, David; University of Southampton<br>Jones, Christine; University of Southampton<br>Read, Robert; University of Southampton
**Primary Subject Heading**:	Infectious diseases
Secondary Subject Heading:	Research methods
Keywords:	Paediatric infectious disease & immunisation < PAEDIATRICS, MICROBIOLOGY, Respiratory infections < THORACIC MEDICINE

SCHOLARONE™
Manuscripts

Full title: Controlled human infection with *Neisseria lactamica* in late pregnancy to measure horizontal transmission and microbiome changes in mother-neonate pairs: a single-arm interventional pilot study protocol

Running title: Protocol for *N. lactamica* controlled human infection in pregnancy

Authors

Anastasia A Theodosiou[1], Jay R Laver[1], Adam P Dale[1], David W Cleary[1], Christine E Jones[1], Robert C Read[1]

Author affiliations

[1]University of Southampton, University Hospital Southampton, Tremona Road, SO166YD Southampton

Corresponding author

Dr. Anastasia A Theodosiou
at1u17@soton.ac.uk
00447748479418
LC72, C Level South Academic Block, University Hospital Southampton, Tremona Road, SO166YD Southampton

For peer review only

Abstract

Introduction: Infant upper respiratory microbiota are derived partly from the maternal respiratory tract, and certain microbiota are associated with altered risk of infections and respiratory disease. *Neisseria lactamica* is a common pharyngeal commensal in young children and is associated with reduced carriage and invasive disease by *Neisseria meningitidis*. Nasal inoculation with *N. lactamica* safely and reproducibly reduces *N. meningitidis* colonisation in healthy adults. We propose nasal inoculation of pregnant women with *N. lactamica*, to establish if neonatal pharyngeal colonisation occurs after birth, and to characterise microbiome evolution in mother-infant pairs over one month post-partum.

Methods and analysis: 20 healthy pregnant women will receive nasal inoculation with *N. lactamica* (wild type strain Y92-1009) at 36-38 weeks gestation. Upper respiratory samples, as well as optional breastmilk, umbilical cord blood and infant venous blood samples, will be collected from mother-infant pairs over one month post-partum. We will assess safety, *N. lactamica* colonisation (by targeted polymerase chain reaction) and longitudinal microevolution (by whole genome sequencing), and microbiome evolution (by 16S rRNA gene sequencing).

Ethics and dissemination: This study has been approved by the London Central Research Ethics Committee (21/PR/0373). Findings will be published in peer-reviewed open-access journals as soon as possible.

Trial registration number (ClinicalTrials.gov): NCT04784845

For peer review only - http://bmjopen.bmj.com/site/about/guidelines.xhtml

Keywords

Controlled human infection; *Neisseria lactamica*; microbiome; respiratory; neonatal; pregnancy

Article summary: Strengths and limitations of this study

1. The *Neisseria lactamica* controlled human infection model is well-characterised, with safety, dosing, immunological and genomic data from over 400 adult participants to date.
2. This is the first prospective study of horizontal respiratory commensal acquisition in neonates following controlled human infection in pregnancy.
3. Longitudinal sampling of multiple respiratory niches in mother-infant pairs allows for microbiome characterisation over time.
4. The small sample size may be insufficient to demonstrate a significant association between *N. lactamica* inoculation and microbiome changes.
5. No causal association between *N. lactamica* inoculation and observed microbiome changes can be concluded in the absence of a control group.

For peer review only

Acronyms / abbreviations used

- CFU – colony-forming units
- GM – genetically modified
- GMP – Good manufacturing practice
- HRA – Health Research Authority
- NHS – National Health Service
- PCR – polymerase chain reaction
- REC – Research Ethics Committee
- rRNA – ribosomal ribonucleic acid
- RTI – respiratory tract infection
- URT – upper respiratory tract
- X-gal - 5-bromo-4-chloro-3-indolyl-β-D-galactopyranoside

Introduction

Upper respiratory pathobionts and infant disease

Upper respiratory tract (URT) pathobionts are common colonising bacteria capable of causing disease in immunocompetent individuals (e.g. *Neisseria meningitidis, Streptococcus pneumoniae, Haemophilus influenzae*). Although harmless in many hosts, these remain a leading cause of global childhood death and disease, especially in the first year of life: globally, 492,000 and 89,000 infants die each year due to respiratory tract infection (RTI) and meningitis, respectively[1]. While many infant infections are vaccine-preventable, global access to and acceptance of vaccines remains incomplete, such that 1.5 million vaccine-preventable childhood deaths occur each year[2]. Furthermore, existing vaccines do not protect against all important childhood respiratory pathogens (such as respiratory syncytial virus and some serotypes of *S. pneumoniae*), and vaccinations are not routinely given in the first two months post-partum, when risk of invasive infection is highest. Rising global antimicrobial resistance compounds these challenges, with 214,000 neonates dying of resistant infections each year[3].

For peer review only - http://bmjopen.bmj.com/site/about/guidelines.xhtml

Recent research has highlighted the relationship between URT pathobionts, childhood disease, and the evolving infant microbiome (the site-specific total microbial community)[4]. Taxonomic profiling (e.g. by bacterial 16S rRNA gene sequencing) demonstrates distinct URT microbiota during acute RTI, and that URT microbiome perturbation is associated with RTI severity and recurrence, childhood chronic wheeze, and early allergic sensitisation[5-8].

Infant RTIs are often preceded by microbiome changes, including increased relative abundance of URT pathobionts and loss of topography (blurring of distinction between microbiomes at adjacent anatomical sites)[9], suggesting a possible causal role in developing infection. However, as up to 93% of infants are colonised with at least one URT pathobiont at any time[10], pathobiont colonisation alone is not necessarily associated with infection: rather, the presence of pathobionts may indicate a state of dysbiosis (microbial dysregulation), in which disease-causing bacteria and viruses can cause infection in a previously resilient ecological niche.

Longitudinal cohort microbiome studies suggest that infant URT flora are acquired at least in part from their mothers: there is greater overlap between a neonate's oral flora and that of its own mother than unrelated mothers, and shared bacterial strains (detected by whole genome sequencing) are more long-lived in an infant's mouth than non-maternal strains[11]. Moreover, a subset of maternal oral strains account for a disproportionately large share of their infants' oral flora[11].

Can upper respiratory microbiota be manipulated to prevent infant disease?

In the future, it is possible that simple methods of modifying the infant nasopharyngeal microbiome could provide useful adjunct protection in the early months of life[12]. Previously, neonatal nasal inoculation with low-virulence *Staphylococcus aureus* strain 502A has been shown to reduce colonisation by high-virulence *S. aureus* strain 80/81 in response to an outbreak[13], while pharyngeal

For peer review only - http://bmjopen.bmj.com/site/about/guidelines.xhtml

inoculation with strain 215 alpha-haemolytic streptococci reduced pharyngeal pathobionts (*Escherichia coli, Klebsiella pneumoniae, S. aureus*) in neonatal intensive care inpatients[14]. Furthermore, a variety of probiotics (living microorganisms administered to confer a health benefit) have been trialled in children and adults, with some reporting a reduction in upper RTI incidence and duration[15]. However, high quality efficacy and microbiome data is lacking, with significant inter-study variation in the bacterial strain (usually lactobacilli, streptococci and bifidobacteria), preparation (usually capsules or dairy supplement), dose and regimen. That being said, there is evidence that many probiotics are safe in pregnancy, neonates and lactating mothers, with some already available over-the-counter to these groups[16].

Controlled human infection with *Neisseria lactamica*

Unlike probiotic studies (which often employ proprietary food supplements with uncontrolled concentrations of bacteria), controlled human infection offers a robust model for studying the impact of a defined bacterial inoculum on a human host. Our research group has previously developed a safe and reliable controlled human infection model using nasal inoculation with 10^4-10^5 colony-forming units (CFU) *N. lactamica* strain Y92-1009 in healthy adults[17,18]. In randomised blinded placebo-controlled studies, we have characterised *N. lactamica* colonisation density, duration, cellular and humoral immune responses, and genomic microevolution[19], observing colonisation rates of up to 85% and no serious adverse reactions following over 400 inoculations.

N. lactamica is a common non-capsulated pharyngeal commensal that does not cause invasive disease in immunocompetent hosts. Colonisation peaks at over 40% in 1-2 year old children[20], before falling to less than 10% in adulthood[21], and *N. lactamica* carriage correlates inversely with *N. meningitidis* colonisation[22]. Indeed, *N. lactamica* controlled human infection in healthy adults reduced naturally-acquired *N. meningitidis* carriage from 18% to 8% (both by displacing existing colonisation and preventing new acquisition), exceeding the typical impact of glycoconjugate ACWY vaccination[17]. This displacement effect is not

For peer review only - http://bmjopen.bmj.com/site/about/guidelines.xhtml

specific to a particular serogroup, unlike available glycoconjugate meningococcal vaccines. Furthermore, natural colonisation with *N. lactamica* has been observed to correlate inversely with invasive meningococcal disease[21].

Rationale for this study

The aim of this study is to establish if nasal inoculation with *N. lactamica* in pregnancy results in neonatal colonisation after birth, and to characterise the impact on the developing neonatal URT microbiome. If successful, this will provide a novel model for inducing and studying person-to-person commensal acquisition in neonates (unlike traditional controlled human infection models, which capture inoculation-induced colonisation). Looking ahead, this may provide a new strategy for reducing *N. meningitidis* colonisation and disease in neonates, and of modifying neonatal microbiome development with a view to improving health outcomes.

For peer review only

Methods and analysis

Study overview

In this prospective controlled human infection study, 20 healthy pregnant women will receive nasal inoculation with 10^5 CFU *N. lactamica* (wild type strain Y92-1009) at 36+0 to 37+6 weeks gestation (Figure 1). Upper respiratory samples and breastmilk will be collected from mother-infant pairs 1 day, 1 week and 1 month post-partum, as well as an umbilical cord blood sample at delivery and an infant venous blood sample at 1 month post-partum. Safety outcomes and relevant clinical data (including mode of delivery, infant feeding and use of antimicrobials) will be recorded. Samples will be analysed by microbiological culture, targeted polymerase chain reaction (PCR), and sequencing to determine *N. lactamica* Y92-1009 presence, colonisation density, and longitudinal microevolution, as well as 16S rRNA gene sequencing to characterise microbiome evolution in mother-neonate pairs.

Study hypothesis, objective and endpoints

We hypothesise that nasal inoculation of pregnant women with 10^5 CFU *N. lactamica* strain Y92-1009 will result in maternal upper respiratory *N. lactamica* colonisation; and that horizontal transmission of upper respiratory *N. lactamica* will occur from experimentally-colonised women to their infants by 4 weeks post-partum.

This study aims to investigate this hypothesis, and to characterise the safety profile, colonisation kinetics, and microbiome impact of nasal *N. lactamica* inoculation in pregnancy on mother-neonate pairs (Table 1).

***N. lactamica* inoculum**

Vials of lyophilised (freeze-dried) *N. lactamica* (LyoNlac) will be produced and stored under Good Manufacturing Practice (GMP)-like standards at the University

For peer review only - http://bmjopen.bmj.com/site/about/guidelines.xhtml

of Southampton. The strain used for lyophilisation (Y92-1009, sequence type 3493, clonal complex 613) originates from the current GMP-compliant pharmaceutical manufacturing facilities at Public Health England (Porton Down, United Kingdom). Lyophilised inoculum stocks can be stored at 4°C (unlike frozen stocks requiring storage at -80°C). LyoNlac will be reconstituted in phosphate buffered saline (PBS) and diluted to a concentration of 10^5 CFU/mL for inoculation. In our previous work, this dose resulted in *N. lactamica* colonisation in 100% of 10 adults receiving lyophilised inoculum and 85% of 20 adults using frozen inoculum (i.e. 90% overall). The inoculum will be administered immediately following reconstitution and serial dilution, and after no more than 30 minutes' delay; recovery of viable bacteria remains over 70% after 30 minutes incubation in room air (unpublished data).

Recruitment of study volunteers

All potentially eligible healthy adult volunteers in the second and third trimesters of pregnancy will be identified by research midwives using a clinical database, and will be sent an ethically-approved study advertisement and a Participant Information Sheet. Individuals who express an interest will be invited to attend a screening visit. Participants will be offered reimbursement for their time, travel and inconvenience.

Study visits and procedures

Screening

Interested individuals will be reviewed by the study doctor at a screening visit (34+0 to 36+6 weeks gestation), to discuss the study timeline and procedures (Table 2) and assess eligibility (Table 3). Eligible individuals who provide informed consent at screening will be considered enrolled. A detailed medical history will be taken, followed by pharyngeal and saliva sampling to assess for *N. lactamica* carriage; enrolled volunteers who are already naturally colonised with *N. lactamica* will not be inoculated. However, they will be invited to remain in the

For peer review only - http://bmjopen.bmj.com/site/about/guidelines.xhtml

study as an opportunistic comparison cohort, and will complete all post-partum visits and sampling as for the inoculated mother-neonate pairs (Table 2).

Inoculation

Eligible consenting volunteers who are not already naturally colonised with *N. lactamica* will receive nasal inoculation with 10^5 CFU *N. lactamica* Y92-1009, using a sterile pipette (0.5mL inoculum per nostril).

Post-partum follow-up

Mother-neonate pairs will be reviewed one day, one week and one month post-partum. At each visit, maternal consent for neonatal sampling will be re-affirmed, and respiratory samples will be collected (Table 2). An optional umbilical cord blood sample (Visit 3) and neonatal venous blood sample (Visit 5) will be collected, as well as relevant clinical data (including mode of delivery, infant feeding and use of antimicrobials). Optional breastmilk samples will also be collected at each post-partum visit (up to 5mL each at Visits 3 to 5). Volunteers may decline optional blood and breastmilk sampling without affecting their enrolment in or completion of the rest of the study.

Volunteers' experiences of participation (including acceptability and tolerability of inoculation and maternal and infant sampling) will be captured using optional questionnaires, to help us identify areas for improving volunteer experience in current and future studies.

Optional Visit 6 (15+/-2 weeks post-partum)

At the time of writing, a study amendment is in progress to include an optional study visit at 15+/-2 weeks post-partum (Visit 6). This visit will involve maternal and infant pharyngeal, saliva and venous blood sampling, as well as saliva and oropharyngeal swabs from household contacts under 5 years old. In a recent *N. lactamica* controlled human infection study involving non-pregnant adults, anti-*N. lactamica* immunoglobulin G (IgG) (peak titre and titre increment) correlated inversely with subsequent pharyngeal colonisation density at 28 days post-inoculation (unpublished data), suggesting that IgG may play a role in controlling

For peer review only - http://bmjopen.bmj.com/site/about/guidelines.xhtml

N. lactamica carriage. Total IgG is up to 1.5 times more concentrated in umbilical cord blood compared with maternal blood[23], and the half-life of transplacental maternal IgG is approximately 30 days[24]; thus, infant anti-*N. lactamica* IgG is predicted to be less than 10-20% that of maternal IgG by 15+/-2 weeks (3 to 4 half-lives). Visit 6 therefore offers a valuable opportunity to investigate the association between anti-*N. lactamica* IgG and *N. lactamica* carriage in mother-infant pairs.

Sample processing

N. lactamica identification

Working in a class 2 microbiological safety cabinet in a Containment Level 2 laboratory, all respiratory samples will be suspended in 1mL storage medium (10% glycerol in 0.1% diethylpyrocarbonate [DEPC]-treated water). Agar selective for *N. lactamica* (GC agar supplemented with 5-bromo-4-chloro-3-indolyl-β-galactopyranoside [X-gal]; Thermo Fisher Scientific, Basingstoke, United Kingdom) will be inoculated with sample solution, and all remaining sample solution will be split into 200μL aliquots for storage in a secure, temperature-monitored -80°C freezer. Following 24-48 hours incubation, colonies with typical *N. lactamica* morphology (blue on GC X-gal agar) will undergo identification by oxidase reaction, Gram stain, microscopy, and analytical profile index (API-NH, bioMérieux, Lyon, France) or matrix-assisted laser desorption/ionization time-of-flight (MALDI-TOF; Bruker, Coventry, United Kingdom). *N. lactamica* density will be quantified and isolates will be stored at -80°C. Breastmilk will also be plated onto selective agar to examine for evidence of *N. lactamica*, and residual samples will be stored at -80°C for microbiome analysis.

N. lactamica Y92-1009 strain-specific PCR

We have identified two chromosomal loci present in *N. lactamica* strain Y92-1009 that, when amplified in conventional multiplex PCR and analysed by agarose gel electrophoresis, produce amplicons with a characteristic band pattern[18]. Using the same multiplex PCR on isolates of naturally circulating strains of *N. lactamica*, this band pattern has not been reproduced. A third locus, present in all known strains

For peer review only - http://bmjopen.bmj.com/site/about/guidelines.xhtml

of *N. lactamica*, is included to differentiate between PCR failure (no bands produced) and any non-Y92-1009 strain of *N. lactamica* (band produced, but Y92-1009-specific bands pattern not present). This PCR assay has been validated using genomic DNA and boiled bacterial lysate as a template, with an optimum annealing temperature 65°C and extension time 15 seconds (unpublished data).

Microbiome analysis

Paired mother-infant frozen sample aliquots will be transported to the University of Edinburgh Centre for Inflammation Research for 16S rRNA gene sequencing[6]. Resulting sequence reads will undergo taxonomic classification to compare mothers and infants, and (assuming some neonates become colonised with *N. lactamica*) colonised and uncolonised infants, across different sample niches and study time points.

***N. lactamica* sequencing**

If any neonates become colonised with *N. lactamica*, paired mother-infant *N. lactamica* isolates will undergo whole genome sequencing to assess for differences in *N. lactamica* microevolution in mother-infant pairs. Resulting sequences will be mapped to a closed *N. lactamica* Y92-1009 reference genome for comparison.

Sample size

To our knowledge, this proof-of-concept represents the first respiratory controlled human infection study in pregnancy. As such, it is not possible to accurately define the sample size needed to conclusively rule out induced horizontal transfer of *N. lactamica*. A pragmatic sample size of 20 inoculated volunteers has been selected: based on our earlier work, *N. lactamica* colonisation is expected in 18 (90%) of volunteers inoculated with 10^5 CFU *N. lactamica*. This sample would therefore fail to detect horizontal transfer only if it occurs in less than 1 in 18 infants (<6%), in which case it would be impractical and even unethical to pursue horizontal *N. lactamica* controlled human infection as a method of studying and manipulating infant commensalisation. By one month of

For peer review only

For peer review only - http://bmjopen.bmj.com/site/about/guidelines.xhtml

age, 2.3% of infants naturally carry *N. lactamica*[17]. Based on a plot of lower confidence limits against observed infant *N. lactamica* colonisation for a sample of 18 experimentally-colonised volunteers, infant *N. lactamica* colonisation greater than 22% (95% CI [2.3%, 41.7%]) would demonstrate that inoculation-induced carriage exceeds natural *N. lactamica* carriage (Figure 2).

We expect to screen at least 24 volunteers to reach our target of 20 inoculations, allowing for 10% dropout and 10% baseline natural *N. lactamica* carriage[18]. It is not clear how maternal or neonatal antibiotics may influence *N. lactamica* carriage and microbiota. At the Principal Investigator's discretion, an additional volunteer may be enrolled and inoculated for every volunteer (or neonate) who receives antibiotics after inoculation, up to a maximum of 10 additional volunteers.

Statistical analysis

The primary endpoint will be expressed as a proportion N/M, where:

- M = Number of mothers colonised with *N. lactamica;*
- N = Number of neonates (aged 0-31 days) colonised with the same strain of *N. lactamica* as their own mother;
- "Colonised" = evidence of *N. lactamica* by culture of upper respiratory swabs (using selective media and confirmation by API or MALDI-TOF) on at least one visit;
- "Same strain" = confirmed using Y92-specific *N. lactamica* PCR for inoculated volunteers, or whole genome sequencing for volunteers already naturally carrying *N. lactamica* at screening.

Subgroup analysis will be performed, to compare inoculated mother-infant pairs with naturally colonised mother-infant pairs, and to compare pairs where the mother or infant received antibiotics with pairs not receiving antibiotics.

Specific bioinformatic analysis strategies will depend on the quantity and quality of genomic data produced during the study, and on the rates of *N. lactamica* colonisation observed. Broadly speaking, microbiome bioinformatic pipelines

For peer review only - http://bmjopen.bmj.com/site/about/guidelines.xhtml

(e.g. QIIME2 and R packages) will be used to perform taxonomic classification, calculation of alpha and beta diversity, and data visualisation.

Patient and public involvement

During early study design, an interview-based Patient and Public Involvement (PPI) project was conducted with 12 pregnant women (unpublished data). All 12 women expressed approval that the proposed study should go ahead, 11 of whom would personally have liked to participate. These discussions led to changes in study design (such as offering follow-up visits in the volunteer's own home, and obtaining an umbilical cord rather than an infant venous blood sample at birth). During participant recruitment following study completion, the University of Southampton Public Engagement Research Unit will assist in showcasing the study at public engagement events.

<u>Ethics and dissemination</u>

Ethics and safety

Volunteer safety and ethical study conduct is of paramount importance. This study has been approved by the NHS Research Ethics committee (REC) and the Health Research Authority (HRA), and is sponsored by the University of Southampton. An External Safety Committee has been appointed to provide independent safety oversight. The *N. lactamica* Y92-1009 inoculum safety profile is well-characterised: over 400 adults have previously been inoculated with this *N. lactamica* strain (of whom 30 received lyophilised inoculum, and 54 received 10^5 CFU), with no serious adverse reactions reported to date[15] (unpublished data). Due to the use of yeast-based and soya-based products in inoculum production, individuals reporting allergies to yeast or soya will not be eligible for enrolment. Inoculation will take place in a clinical area with access to adult, obstetric and paediatric emergency services. Volunteers will be reviewed by a study doctor immediately prior to and 30 minutes after inoculation.

For peer review only - http://bmjopen.bmj.com/site/about/guidelines.xhtml

Adverse events will be recorded throughout the study, and volunteers will be encouraged to report any concerns (including by telephone to the study doctor out-of-hours). Safety data will also be collected from any inoculated withdrawn volunteers, by telephone review at four weeks post-partum. As per the current Good Clinical Practice guideline[25], adverse events are any untoward medical occurrence in a volunteer or their neonate, temporally, but not necessarily causally, related to study participation. Delivery of routine peripartum care is not considered an adverse event. Serious adverse events include hospitalisation (or prolongation of hospitalisation for routine peripartum care), persistent or significant disability, a life-threatening occurrence, or death from any cause.

Mitigation strategies are in place to minimise the risk of SARS-CoV2 transmission to participants and study staff. Where possible, study visits will be conducted in the volunteer's home, practicing social distancing and appropriate personal protective equipment for non-aerosol generating procedures. If participants report symptoms of or contact with anyone with suspected or confirmed SARS-CoV2 infection, study visits will be rescheduled until testing or self-isolation as necessary.

Data management and dissemination

All data will be stored in link-anonymised format using paper case report forms stored securely at the research site. Fully anonymised sequencing data will be uploaded to a curated online data repository. Research findings will be published in peer-reviewed journals as soon as is practicable, with an online link to the final approved study protocol. Participants will be provided with a lay summary of the study results once available.

Potential benefits and study impact

To our knowledge, this is the first respiratory controlled human infection study in pregnancy, and, if successful, would be the first to demonstrate induced person-to-person commensal transmission. This novel model could be used to

For peer review only - http://bmjopen.bmj.com/site/about/guidelines.xhtml

characterise microbiome and immunological changes associated with induced commensalisation. Inducing *N. lactamica* colonisation in neonates may also provide a new strategy for reducing *N. meningitidis* colonisation and even disease in infants. Although many neonates are protected by trans-placental maternal antibodies, invasive meningococcal disease does rarely occur in infants, especially before vaccination[26]. Looking ahead, improved understanding of microbiome evolution following controlled human infection is necessary before inoculation in neonates (rather than in pregnant adults) can be considered.

Our team have recently trialled nasal inoculation in healthy non-pregnant adults using genetically-modified (GM) *N. lactamica* that expresses the meningococcal antigen *Neisseria* adhesin A (NadA)[27]. This GM inoculum has proved safe and effective at eliciting immune responses to NadA, providing a proof-of-concept that a harmless commensal may be used as a vehicle for prolonged mucosal exposure to an antigen of interest. Looking ahead, GM *N. lactamica* expressing antigens from other clinically-important paediatric respiratory pathogens could be explored, to complement existing childhood vaccination.

For peer review only

Tables

Primary endpoint	Secondary endpoints
1. Confirmation of neonatal (0-31 days) *N. lactamica* colonisation by selective culture of nasopharyngeal and saliva samples.	1. Confirmation of neonatal *N. lactamica* Y92-1009 colonisation by strain-specific PCR.<br>2. *N. lactamica* colonisation density in inoculated volunteers compared with their infants across study time points and sample niche.<br>3. Microbiome composition across study time points and sample niche in inoculated volunteers compared with their infants, and in colonised compared with uncolonised infants.<br>4. *N. lactamica* genome sequence for isolates derived from inoculated volunteers compared with their infants across study time points and sample niche.
**Safety endpoints**<br>1. Percentage of inoculated volunteers with adverse reactions or serious adverse events within the study period.<br>2. Percentage of neonates with serious adverse events within the study period.	

Table 1. Primary, secondary and safety study endpoints. PCR: polymerase chain reaction.

	Screening (Visit 1)	Inoculation (Visit 2)	Birth (Visit 3)	Follow-up (Visits 4 and 5)		Optional (Visit 6)
**Timeline**	**34+0 to 36+6 weeks gestation**	**36+0 to 37+6 weeks gestation**	**0-24 hours post-partum**	**7 +/- 3 days post-partum**	**28 +/- 3 days post-partum**	**15 +/- 2 weeks post-partum**
**Participant Information Sheet and consent form**	+					
**Clinical review**	+	+	+	+	+	+
***N. lactamica* nasal inoculation**		+ (Unless already colonised)				
**Nasopharyngeal swabs (mother)**	+	+	+	+	+	+
**Oropharyngeal swabs (mother)**	+	+	+	+	+	+
**Saliva swabs (mother)**	+	+	+	+	+	+
**Nasopharyngeal swabs (infant)**			+	+	+	+

For peer review only - http://bmjopen.bmj.com/site/about/guidelines.xhtml

**Saliva swabs (infant)**			+	+	+	+
**Umbilical cord blood**			Optional			
**Infant venous blood (2ml)**					Optional	Optional
**Maternal breast milk**			Optional	Optional	Optional	Optional
**Maternal venous blood (5ml)**						Optional
**Oropharyngeal & saliva swabs (under 5 years)**						Optional

Table 2: Study visits and activities.

Inclusion criteria (all must apply):

- Healthy adult aged 18 years or over on the day of enrolment.
- Singleton pregnancy, 34+0 to 36+6 weeks gestation on the day of enrolment.
- Documentation of a 20-week ultrasound scan with no life-limiting congenital anomalies, and no maxillofacial, otorhinolaryngolocial or neuroanatomical anomalies.
- Able and willing (in the Investigator's opinion) to comply with all study requirements.
- Able and willing to give written informed consent to participate in the study.
- Booked to receive antenatal care at University Hospital Southampton NHS Foundation Trust (including any associated community antenatal care facilities), United Kingdom.

Exclusion criteria (none must apply):

- Any confirmed or suspected immunosuppressive or immunocompromised state, including: HIV infection; asplenia; recurrent severe infections; or use of immunosuppressant medication (for more than 14 days within the past 6 months, excluding topical and inhaled steroids).
- Planned use of immunosuppressant medication in later pregnancy or post-partum.

For peer review only

For peer review only - http://bmjopen.bmj.com/site/about/guidelines.xhtml

- Occupational, household or intimate contact with any immunosuppressed persons.
- Participation within the last 12 weeks in a clinical trial involving receipt of an investigational product, or planned use of an investigational product during the study period.
- Prior participation at any time in research studies involving inoculation with *N. lactamica.*
- Use of oral or intravenous antibiotics within 30 days prior to the inoculation visit.
- Planned use of oral or intravenous antibiotics at any time during the study period (e.g. for planned elective caesarean section or group B streptococcus colonisation).
- Allergy to soya or yeast.
- Previous stillbirth or neonatal death.
- Pre-pregnancy diabetes mellitus.
- Any other finding that may (in the Investigator's opinion): increase the risk to the volunteer (or their fetus/infant or close contacts) of participating in the study; affect the volunteer's ability to participate in the study and complete follow-up; or impair interpretation of study data.

Table 3. Inclusion and exclusion criteria. NHS: National Health Service; HIV: human immunodeficiency virus.

For peer review only

For peer review only - http://bmjopen.bmj.com/site/about/guidelines.xhtml

References

1. Roser M et al. Child and Infant Mortality. OurWorldInData.org Nov 2019. Retrieved Dec 2020 from: https://ourworldindata.org/child-mortality.
2. Gostin L et al. The public health crisis of underimmunisation: a global plan of action. Lancet Infect Dis 2020;20(1):e11-16.
3. Laxminarayan R et al. Access to effective antimicrobials: a worldwide challenge. Lancet 2016;387(10014):168-75.
4. Theodosiou AA et al. The infant pharyngeal microbiomes: origin, impact and manipulation. Curr Opin Infect Dis 2020;33(6):548-555.
5. Teo S et al. Dynamics of airway microbiota identify a critical window for interplay of pathogenic bacteria and allergic sensitization in childhood respiratory disease. Cell Host Microbe 2018;24(3):341–352.
6. Luna P et al. The association between anterior nares and nasopharyngeal microbiota in infants hospitalized for bronchiolitis. Microbiome 2018; 6(1):2.
7. Bosch A et al. Maturation of the infant respiratory microbiota, environmental drivers, and health consequences.Am J Respir Crit Care Med 2017;196(12):1582–1590.
8. Thorsen J et al. Infant airway microbiota and topical immune perturbations in the origins of childhood asthma. Nat Commun 2019;10(1):5001.
9. Man W et al. Loss of microbial topography between oral and nasopharyngeal microbiota and development of respiratory infections early in life. Am J Respir Crit Care Med 2019;200(6):760–770.
10. de Steenhuijsen Piters W et al. The role of the local microbial ecosystem in respiratory health and disease. Philos Trans R Soc B Biol Sci 2015;370(1675):20140294.
11. Ferretti P et al. Mother-to-infant microbial transmission from different body sites shapes the developing infant gut microbiome. Cell Host Microbe 2018;24(1):133–145.
12. Theodosiou AA et al. Manipulating the infant respiratory microbiomes to improve clinical outcomes: A review of the literature. J Infect 2021;82(6):247-252.
13. Shinefield H et al. Bacterial interference: its effect on nursery-acquired infection with *Staphylococcus aureus*. V. An analysis and interpretation. Am J Dis Child 1963;105:683–8.
14. Sprunt K et al. Abnormal colonization of neonates in an ICU: conversion to normal colonization by pharyngeal implantation of alpha haemolytic *Streptococcus* strain 215. Pediatr Res 1980;14(4):308–313.
15. Hao Q et al. Probiotics for preventing acute upper respiratory tract infections. Cochrane Database Syst Rev 2015;(2):CD006895.
16. Elias J et al. Are probiotics safe for use during pregnancy & lactation? Can Fam Physician 2011;57(3):299-301.
17. Deasy A et al. Nasal Inoculation of the Commensal *Neisseria lactamica* Inhibits Carriage of *Neisseria meningitidis* by Young Adults. Clin Infect Dis 2015;60(10):1512-20.
18. Dale AP et al. *Neisseria lactamica* Controlled Human Infection Model. Methods in Molecular Biology (Clifton, N.J.) 2022 ;2414:387-404.
19. Pandey A et al. Microevolution of *Neisseria lactamica* during nasopharyngeal colonisation induced by controlled human infection. Nat Commun 2018;9(1):4753.
20. Bennett J et al. Genetic diversity and carriage dynamics of *Neisseria lactamica* in infants. Infect Immun 2005;73(4):2424.

For peer review only

21. Olsen S et al. Pharyngeal carriage of *N meningitidis* and *N lactamica* in households with infants within areas with high and low incidences of meningococcal disease. Epidemiol Infect 1991;106(3):445-57.
22. Gold R et al. Carriage of *N meningitidis* and *N lactamica* in infants and children. J Infect Dis 1978;137(2):112.
23. Clements T et al. Update on Transplacental Transfer of IgG Subclasses: Impact of Maternal and Fetal Factors. Front Immunol 2020;11:1920.
24. Oguti B et al. The half-life of maternal transplacental antibodies against diphtheria, tetanus, and pertussis in infants: an individual participant data meta-analysis. Vaccine 2022;40(3):450-458.
25. International Conference on Harmonisation E6 Good Clinical Practice CPMP/ICH/135/95. Retrieved Feb 2022 from: https://www.ich.org/page/efficacy-guidelines#6-2.
26. Kiray Bas et al. Neonatal infection with *Neisseria meningitidis*: analysis of a 97-year period plus case study. J Clin Microbiol 2014;52(9):3478-82.
27. Laver JR et al. A recombinant commensal bacteria elicits heterologous antigen-specific immune responses during pharyngeal carriage. Sci Transl Med. 2021;13(601).

<u>Contributorship statement</u>

A Theodosiou prepared the manuscript, led the study design, and is the Principal Investigator and overall guarantor for the study. C Jones and R Read are the Chief Investigators and provided clinical and academic supervision. A Dale was involved in clinical and microbiological study design, D Cleary supervised genomic and bioinformatic study design, and J Laver supervised laboratory and molecular study design. All authors reviewed, edited and approved the final manuscript.

<u>Funding statement</u>

This work is supported by a Medical Research Council Clinical Research Training Fellowship (grant number MR/V002015/1) awarded to Dr Anastasia A Theodosiou. Robert C Read is an NIHR Senior Investigator. The funders have not had input into the design or execution of the study or the preparation of this manuscript.

<u>Competing interests statement</u>

The authors have no competing interests to declare.

For peer review only

Additional trial registration and SPIRIT checklist data

Primary trial registration number (ClinicalTrials.gov): NCT04784845 (registered 5th March 2021)
Secondary identifying numbers: IRAS (NHS REC) 290865, ERGO (Sponsor) 61640
Date of first enrolment: 25th October 2021
Recruitment status: recruiting
Research site: University Hospital Southampton NHS Foundation Trust, Tremona Road, Southampton SO166YD, United Kingdom
Current protocol version: Version 1.3 (22nd June 2021). At the time of writing, Protocol Version 1.4 (4th February 2022) is being reviewed by the Research Ethics Committee as part of a substantial study amendment.
Contact for public and scientific queries: Dr A Theodosiou (PI) at1u17@soton.ac.uk
Contact for study Sponsor: University of Southampton, rgoinfo@soton.ac.uk
Sponsor insurance statement: A specialist insurance policy is in place, which would operate in the event of any volunteer suffering harm as a result of involvement in the study.
Data monitoring: To be overseen by the Sponsor. For a small pilot study investigating basic science rather than clinical outcomes, a formal external Data Monitoring Committee was not felt to be appropriate by the Sponsor or the Research Ethics Committee.

For peer review only

Figure legends

Figure 1: Study overview flowchart. Nlac: *Neisseria lactamica*; m: months; w: weeks; h: hours; API: analytical profile index; MALDI [MALDI-TOF]: matrix-assisted laser desorption/ionization time-of-flight; CFU: colony forming units; PCR: polymerase chain reaction; DNA: deoxyribonucleic acid

Figure 2: Lower confidence limit plot for inoculation-induced *N. lactamica* (Nlac) colonisation.

For peer review only

For peer review only - http://bmjopen.bmj.com/site/about/guidelines.xhtml

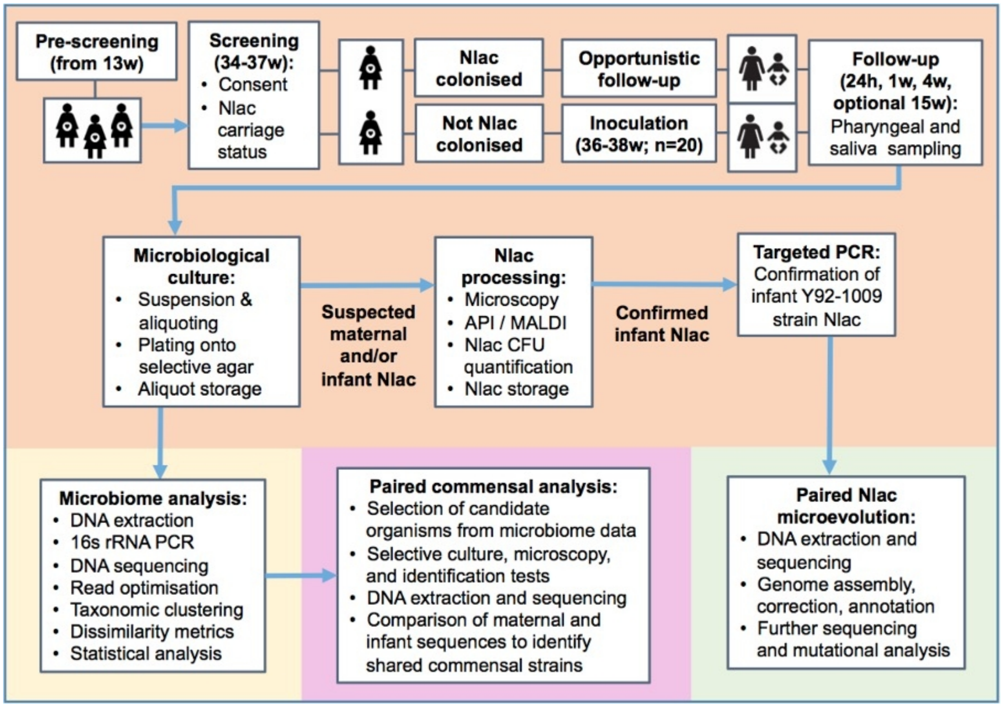

Figure 1: Study overview flowchart. Nlac: Neisseria lactamica; m: months; w: weeks; h: hours; API: analytical profile index; MALDI [MALDI-TOF]: matrix-assisted laser desorption/ionization time-of-flight; CFU: colony forming units; PCR: polymerase chain reaction; DNA: deoxyribonucleic acid

159x111mm (144 x 144 DPI)

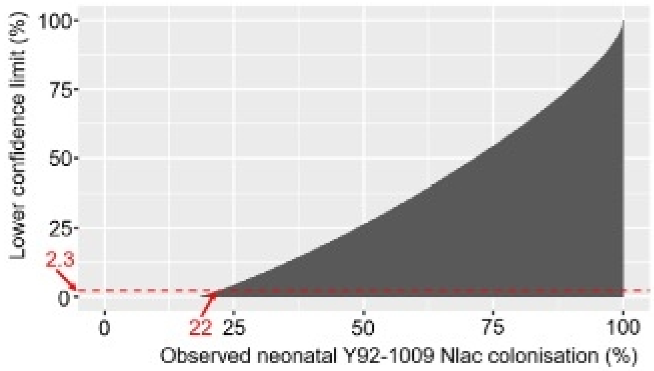

Figure 2: Lower confidence limit plot for inoculation-induced N. lactamica (Nlac) colonisation.

116x66mm (72 x 72 DPI)

SPIRIT 2013 Checklist: Recommended items to address in a clinical trial protocol and related documents*

Section/ Item	Item No	Description	Page/ Line
**Administrative information**			
Title	1	Descriptive title identifying the study design, population, interventions, and, if applicable, trial acronym	1/1
Trial registration	2a	Trial identifier and registry name. If not yet registered, name of intended registry	22/559
	2b	All items from the World Health Organization Trial Registration Data Set	22/557
Protocol version	3	Date and version identifier	22/565
Funding	4	Sources and types of financial, material, and other support	21/545
Roles and responsibilities	5a	Names, affiliations, and roles of protocol contributors	1/9
	5b	Name and contact information for the trial sponsor	22/570
	5c	Role of study sponsor and funders, if any, in study design; collection, management, analysis, and interpretation of data; writing of the report; and the decision to submit the report for publication, including whether they will have ultimate authority over any of these activities	21/549
	5d	Composition, roles, and responsibilities of the coordinating centre, steering committee, endpoint adjudication committee, data management team, and other individuals or groups overseeing the trial, if applicable (see Item 21a for data monitoring committee)	N/A
**Introduction**			
Background and rationale	6a	Description of research question and justification for undertaking the trial, including summary of relevant studies (published and unpublished) examining benefits and harms for each intervention	7/169
	6b	Explanation for choice of comparators	17/462
Objectives	7	Specific objectives or hypotheses	8/197

For peer review only

Trial design	8	Description of trial design including type of trial (eg, parallel group, crossover, factorial, single group), allocation ratio, and framework (eg, superiority, equivalence, exploratory)	8/182
**Methods: Participants, interventions, and outcomes**			
Study setting	9	Description of study settings (eg, community clinic, academic hospital) and list of countries where data will be collected. Reference to where list of study sites can be obtained	18/467
Eligibility criteria	10	Inclusion and exclusion criteria for participants. If applicable, eligibility criteria for study centres and individuals who will perform the interventions (eg, surgeons, psychotherapists)	18/467
Interventions	11a	Interventions for each group with sufficient detail to allow replication, including how and when they will be administered	8/209
	11b	Criteria for discontinuing or modifying allocated interventions for a given trial participant (eg, drug dose change in response to harms, participant request, or improving/worsening disease)	N/A
	11c	Strategies to improve adherence to intervention protocols, and any procedures for monitoring adherence (eg, drug tablet return, laboratory tests)	N/A
	11d	Relevant concomitant care and interventions that are permitted or prohibited during the trial	N/A
Outcomes	12	Primary, secondary, and other outcomes, including the specific measurement variable (eg, systolic blood pressure), analysis metric (eg, change from baseline, final value, time to event), method of aggregation (eg, median, proportion), and time point for each outcome. Explanation of the clinical relevance of chosen efficacy and harm outcomes is strongly recommended	17/462
Participant timeline	13	Time schedule of enrolment, interventions (including any run-ins and washouts), assessments, and visits for participants. A schematic diagram is highly recommended (see Figure)	Figure 1
Sample size	14	Estimated number of participants needed to achieve study objectives and how it was determined, including clinical and statistical assumptions supporting any sample size calculations	12/332
Recruitment	15	Strategies for achieving adequate participant enrolment to reach target sample size	9/226

For peer review only - http://bmjopen.bmj.com/site/about/guidelines.xhtml

Methods: Data collection, management, and analysis

Data collection methods	18a	Plans for assessment and collection of outcome, baseline, and other trial data, including any related processes to promote data quality (eg, duplicate measurements, training of assessors) and a description of study instruments (eg, questionnaires, laboratory tests) along with their reliability and validity, if known. Reference to where data collection forms can be found, if not in the protocol	9/235
	18b	Plans to promote participant retention and complete follow-up, including list of any outcome data to be collected for participants who discontinue or deviate from intervention protocols	15/411
Data management	19	Plans for data entry, coding, security, and storage, including any related processes to promote data quality (eg, double data entry; range checks for data values). Reference to where details of data management procedures can be found, if not in the protocol	15/428
Statistical methods	20a	Statistical methods for analysing primary and secondary outcomes. Reference to where other details of the statistical analysis plan can be found, if not in the protocol	13/359
	20b	Methods for any additional analyses (eg, subgroup and adjusted analyses)	13/369
	20c	Definition of analysis population relating to protocol non-adherence (eg, as randomised analysis), and any statistical methods to handle missing data (eg, multiple imputation)	N/A

Methods: Monitoring

Data monitoring	21a	Composition of data monitoring committee (DMC); summary of its role and reporting structure; statement of whether it is independent from the sponsor and competing interests; and reference to where further details about its charter can be found, if not in the protocol. Alternatively, an explanation of why a DMC is not needed	22/575
	21b	Description of any interim analyses and stopping guidelines, including who will have access to these interim results and make the final decision to terminate the trial	N/A
Harms	22	Plans for collecting, assessing, reporting, and managing solicited and spontaneously reported adverse events and other unintended effects of trial interventions or trial conduct	15/409

Auditing	23	Frequency and procedures for auditing trial conduct, if any, and whether the process will be independent from investigators and the sponsor	N/A
**Ethics and dissemination**			
Research ethics approval	24	Plans for seeking research ethics committee/institutional review board (REC/IRB) approval	2/43
Protocol amendments	25	Plans for communicating important protocol modifications (eg, changes to eligibility criteria, outcomes, analyses) to relevant parties (eg, investigators, REC/IRBs, trial participants, trial registries, journals, regulators)	10/270
Consent or assent	26a	Who will obtain informed consent or assent from potential trial participants or authorised surrogates, and how (see Item 32)	9/237
	26b	Additional consent provisions for collection and use of participant data and biological specimens in ancillary studies, if applicable	N/A
Confidentiality	27	How personal information about potential and enrolled participants will be collected, shared, and maintained in order to protect confidentiality before, during, and after the trial	15/428
Declaration of interests	28	Financial and other competing interests for principal investigators for the overall trial and each study site	21/553
Access to data	29	Statement of who will have access to the final trial dataset, and disclosure of contractual agreements that limit such access for investigators	15/428
Ancillary and post-trial care	30	Provisions, if any, for ancillary and post-trial care, and for compensation to those who suffer harm from trial participation	22/572
Dissemination policy	31a	Plans for investigators and sponsor to communicate trial results to participants, healthcare professionals, the public, and other relevant groups (eg, via publication, reporting in results databases, or other data sharing arrangements), including any publication restrictions	15/428
	31b	Authorship eligibility guidelines and any intended use of professional writers	21/539
	31c	Plans, if any, for granting public access to the full protocol, participant-level dataset, and statistical code	15/428

*It is strongly recommended that this checklist be read in conjunction with the SPIRIT 2013 Explanation & Elaboration for important clarification on the items. Amendments to the protocol should be tracked and dated. The SPIRIT checklist is copyrighted by the SPIRIT Group under the Creative Commons "Attribution-NonCommercial-NoDerivs 3.0 Unported" license.

For peer review only

BMJ Open

Controlled human infection with Neisseria lactamica in late pregnancy to measure horizontal transmission and microbiome changes in mother-neonate pairs: a single-arm interventional pilot study protocol

Journal:	*BMJ Open*
Manuscript ID	bmjopen-2021-056081.R2
Article Type:	Protocol
Date Submitted by the Author:	28-Apr-2022

Complete List of Authors:	Theodosiou, Anastasia; University of Southampton, Clinical and Experimental Sciences<br>Laver, Jay; University of Southampton<br>Dale, Adam; University of Southampton, Clinical and Experimental Sciences<br>Cleary, David; University of Southampton<br>Jones, Christine; University of Southampton<br>Read, Robert; University of Southampton

**Primary Subject Heading**:	Infectious diseases
Secondary Subject Heading:	Research methods
Keywords:	Paediatric infectious disease & immunisation < PAEDIATRICS, MICROBIOLOGY, Respiratory infections < THORACIC MEDICINE

SCHOLARONE™
Manuscripts

Full title: Controlled human infection with *Neisseria lactamica* in late pregnancy to measure horizontal transmission and microbiome changes in mother-neonate pairs: a single-arm interventional pilot study protocol

Running title: Protocol for *N. lactamica* controlled human infection in pregnancy

Authors

Anastasia A Theodosiou[1], Jay R Laver[1], Adam P Dale[1], David W Cleary[1], Christine E Jones[1], Robert C Read[1]

Author affiliations

[1]University of Southampton, University Hospital Southampton, Tremona Road, SO166YD Southampton

Corresponding author

Dr. Anastasia A Theodosiou
at1u17@soton.ac.uk
00447748479418
LC72, C Level South Academic Block, University Hospital Southampton, Tremona Road, SO166YD Southampton

For peer review only

For peer review only - http://bmjopen.bmj.com/site/about/guidelines.xhtml

Abstract

Introduction: Infant upper respiratory microbiota are derived partly from the maternal respiratory tract, and certain microbiota are associated with altered risk of infections and respiratory disease. *Neisseria lactamica* is a common pharyngeal commensal in young children and is associated with reduced carriage and invasive disease by *Neisseria meningitidis*. Nasal inoculation with *N. lactamica* safely and reproducibly reduces *N. meningitidis* colonisation in healthy adults. We propose nasal inoculation of pregnant women with *N. lactamica*, to establish if neonatal pharyngeal colonisation occurs after birth, and to characterise microbiome evolution in mother-infant pairs over one month post-partum.

Methods and analysis: 20 healthy pregnant women will receive nasal inoculation with *N. lactamica* (wild type strain Y92-1009) at 36-38 weeks gestation. Upper respiratory samples, as well as optional breastmilk, umbilical cord blood and infant venous blood samples, will be collected from mother-infant pairs over one month post-partum. We will assess safety, *N. lactamica* colonisation (by targeted polymerase chain reaction) and longitudinal microevolution (by whole genome sequencing), and microbiome evolution (by 16S rRNA gene sequencing).

Ethics and dissemination: This study has been approved by the London Central Research Ethics Committee (21/PR/0373). Findings will be published in peer-reviewed open-access journals as soon as possible.

Trial registration number (ClinicalTrials.gov): NCT04784845

For peer review only - http://bmjopen.bmj.com/site/about/guidelines.xhtml

Keywords

Controlled human infection; *Neisseria lactamica*; microbiome; respiratory; neonatal; pregnancy

Article summary: Strengths and limitations of this study

1. The *Neisseria lactamica* controlled human infection model is well-characterised, with safety, dosing, immunological and genomic data from over 400 adult participants to date.
2. This is the first prospective study of horizontal respiratory commensal acquisition in neonates following controlled human infection in pregnancy.
3. Longitudinal sampling of multiple respiratory niches in mother-infant pairs allows for microbiome characterisation over time.
4. The small sample size may be insufficient to demonstrate a significant association between *N. lactamica* inoculation and microbiome changes.
5. No causal association between *N. lactamica* inoculation and observed microbiome changes can be concluded in the absence of a control group.

For peer review only

For peer review only - http://bmjopen.bmj.com/site/about/guidelines.xhtml

Acronyms / abbreviations used

- CFU – colony-forming units
- GM – genetically modified
- GMP – Good manufacturing practice
- HRA – Health Research Authority
- NHS – National Health Service
- PCR – polymerase chain reaction
- REC – Research Ethics Committee
- rRNA – ribosomal ribonucleic acid
- RTI – respiratory tract infection
- URT – upper respiratory tract
- X-gal - 5-bromo-4-chloro-3-indolyl-β-D-galactopyranoside

Introduction

Upper respiratory pathobionts and infant disease

Upper respiratory tract (URT) pathobionts are common colonising bacteria capable of causing disease in immunocompetent individuals (e.g. *Neisseria meningitidis, Streptococcus pneumoniae, Haemophilus influenzae*). Although harmless in many hosts, these remain a leading cause of global childhood death and disease, especially in the first year of life: globally, 492,000 and 89,000 infants die each year due to respiratory tract infection (RTI) and meningitis, respectively[1]. While many infant infections are vaccine-preventable, global access to and acceptance of vaccines remains incomplete, such that 1.5 million vaccine-preventable childhood deaths occur each year[2]. Furthermore, existing vaccines do not protect against all important childhood respiratory pathogens (such as respiratory syncytial virus and some serotypes of *S. pneumoniae*), and vaccinations are not routinely given in the first two months post-partum, when risk of invasive infection is highest. Rising global antimicrobial resistance compounds these challenges, with 214,000 neonates dying of resistant infections each year[3].

For peer review only

For peer review only - http://bmjopen.bmj.com/site/about/guidelines.xhtml

Recent research has highlighted the relationship between URT pathobionts, childhood disease, and the evolving infant microbiome (the site-specific total microbial community)[4]. Taxonomic profiling (e.g. by bacterial 16S rRNA gene sequencing) demonstrates distinct URT microbiota during acute RTI, and that URT microbiome perturbation is associated with RTI severity and recurrence, childhood chronic wheeze, and early allergic sensitisation[5-8].

Infant RTIs are often preceded by microbiome changes, including increased relative abundance of URT pathobionts and loss of topography (blurring of distinction between microbiomes at adjacent anatomical sites)[9], suggesting a possible causal role in developing infection. However, as up to 93% of infants are colonised with at least one URT pathobiont at any time[10], pathobiont colonisation alone is not necessarily associated with infection: rather, the presence of pathobionts may indicate a state of dysbiosis (microbial dysregulation), in which disease-causing bacteria and viruses can cause infection in a previously resilient ecological niche.

Longitudinal cohort microbiome studies suggest that infant URT flora are acquired at least in part from their mothers: there is greater overlap between a neonate's oral flora and that of its own mother than unrelated mothers, and shared bacterial strains (detected by whole genome sequencing) are more long-lived in an infant's mouth than non-maternal strains[11]. Moreover, a subset of maternal oral strains account for a disproportionately large share of their infants' oral flora[11].

Can upper respiratory microbiota be manipulated to prevent infant disease?

In the future, it is possible that simple methods of modifying the infant nasopharyngeal microbiome could provide useful adjunct protection in the early months of life[12]. Previously, neonatal nasal inoculation with low-virulence *Staphylococcus aureus* strain 502A has been shown to reduce colonisation by high-virulence *S. aureus* strain 80/81 in response to an outbreak[13], while pharyngeal

For peer review only - http://bmjopen.bmj.com/site/about/guidelines.xhtml

inoculation with strain 215 alpha-haemolytic streptococci reduced pharyngeal pathobionts (*Escherichia coli, Klebsiella pneumoniae, S. aureus*) in neonatal intensive care inpatients[14]. Furthermore, a variety of probiotics (living microorganisms administered to confer a health benefit) have been trialled in children and adults, with some reporting a reduction in upper RTI incidence and duration[15]. However, high quality efficacy and microbiome data is lacking, with significant inter-study variation in the bacterial strain (usually lactobacilli, streptococci and bifidobacteria), preparation (usually capsules or dairy supplement), dose and regimen. That being said, there is evidence that many probiotics are safe in pregnancy, neonates and lactating mothers, with some already available over-the-counter to these groups[16].

Controlled human infection with *Neisseria lactamica*

Unlike probiotic studies (which often employ proprietary food supplements with uncontrolled concentrations of bacteria), controlled human infection offers a robust model for studying the impact of a defined bacterial inoculum on a human host. Our research group has previously developed a safe and reliable controlled human infection model using nasal inoculation with 10^4-10^5 colony-forming units (CFU) *N. lactamica* strain Y92-1009 in healthy adults[17,18]. In randomised blinded placebo-controlled studies, we have characterised *N. lactamica* colonisation density, duration, cellular and humoral immune responses, and genomic microevolution[19], observing colonisation rates of up to 85% and no serious adverse reactions following over 400 inoculations.

N. lactamica is a common non-capsulated pharyngeal commensal that does not cause invasive disease in immunocompetent hosts. Colonisation peaks at over 40% in 1-2 year old children[20], before falling to less than 10% in adulthood[21], and *N. lactamica* carriage correlates inversely with *N. meningitidis* colonisation[22]. Indeed, *N. lactamica* controlled human infection in healthy adults reduced naturally-acquired *N. meningitidis* carriage from 18% to 8% (both by displacing existing colonisation and preventing new acquisition), exceeding the typical impact of glycoconjugate ACWY vaccination[17]. This displacement effect is not

For peer review only - http://bmjopen.bmj.com/site/about/guidelines.xhtml

specific to a particular serogroup, unlike available glycoconjugate meningococcal vaccines. Furthermore, natural colonisation with *N. lactamica* has been observed to correlate inversely with invasive meningococcal disease[21].

Rationale for this study

The aim of this study is to establish if nasal inoculation with *N. lactamica* in pregnancy results in neonatal colonisation after birth, and to characterise the impact on the developing neonatal URT microbiome. If successful, this will provide a novel model for inducing and studying person-to-person commensal acquisition in neonates (unlike traditional controlled human infection models, which capture inoculation-induced colonisation). Looking ahead, this may provide a new strategy for reducing *N. meningitidis* colonisation and disease in neonates, and of modifying neonatal microbiome development with a view to improving health outcomes.

For peer review only

Methods and analysis

Study overview

In this prospective controlled human infection study, 20 healthy pregnant women will receive nasal inoculation with 10^5 CFU *N. lactamica* (wild type strain Y92-1009) at 36+0 to 37+6 weeks gestation (Figure 1). Upper respiratory samples and breastmilk will be collected from mother-infant pairs 1 day, 1 week and 1 month post-partum, as well as an umbilical cord blood sample at delivery and an infant venous blood sample at 1 month post-partum. Adverse events and relevant clinical data (including mode of delivery, infant feeding and use of antimicrobials) will be recorded. Samples will be analysed by microbiological culture, targeted polymerase chain reaction (PCR), and sequencing to determine *N. lactamica* Y92-1009 presence, colonisation density, and longitudinal microevolution, as well as 16S rRNA gene sequencing to characterise microbiome evolution in mother-neonate pairs.

Study hypothesis, objective and endpoints

We hypothesise that nasal inoculation of pregnant women with 10^5 CFU *N. lactamica* strain Y92-1009 will result in maternal upper respiratory *N. lactamica* colonisation; and that horizontal transmission of upper respiratory *N. lactamica* will occur from experimentally-colonised women to their infants by 4 weeks post-partum.

This study aims to investigate this hypothesis, and to characterise the safety, colonisation kinetics, and microbiome impact of nasal *N. lactamica* inoculation in pregnancy on mother-neonate pairs (Table 1).

***N. lactamica* inoculum**

Vials of lyophilised (freeze-dried) *N. lactamica* (LyoNlac) will be produced and stored under Good Manufacturing Practice (GMP)-like standards at the University

For peer review only - http://bmjopen.bmj.com/site/about/guidelines.xhtml

of Southampton. The strain used for lyophilisation (Y92-1009, sequence type 3493, clonal complex 613) originates from the current GMP-compliant pharmaceutical manufacturing facilities at Public Health England (Porton Down, United Kingdom). Lyophilised inoculum stocks can be stored at 4°C (unlike frozen stocks requiring storage at -80°C). LyoNlac will be reconstituted in phosphate buffered saline (PBS) and diluted to a concentration of 10^5 CFU/mL for inoculation. In our previous work, this dose resulted in *N. lactamica* colonisation in 100% of 10 adults receiving lyophilised inoculum and 85% of 20 adults using frozen inoculum (i.e. 90% overall). The inoculum will be administered immediately following reconstitution and serial dilution, and after no more than 30 minutes' delay; recovery of viable bacteria remains over 70% after 30 minutes incubation in room air (unpublished data).

Recruitment of study volunteers

All potentially eligible healthy adult volunteers in the second and third trimesters of pregnancy will be identified by research midwives using a clinical database, and will be sent an ethically-approved study advertisement and a Participant Information Sheet. Individuals who express an interest will be invited to attend a screening visit. Participants will be offered reimbursement for their time, travel and inconvenience.

Study visits and procedures

Screening

Interested individuals will be reviewed by the study doctor at a screening visit (34+0 to 36+6 weeks gestation), to discuss the study timeline and procedures (Table 2) and assess eligibility (Table 3). Eligible individuals who provide informed consent at screening will be considered enrolled. A detailed medical history will be taken, followed by pharyngeal and saliva sampling to assess for *N. lactamica* carriage; enrolled volunteers who are already naturally colonised with *N. lactamica* will not be inoculated. However, they will be invited to remain in the

For peer review only - http://bmjopen.bmj.com/site/about/guidelines.xhtml

study as an opportunistic comparison cohort, and will complete all post-partum visits and sampling as for the inoculated mother-neonate pairs (Table 2).

Inoculation

Eligible consenting volunteers who are not already naturally colonised with *N. lactamica* will receive nasal inoculation with 10^5 CFU *N. lactamica* Y92-1009, using a sterile pipette (0.5mL inoculum per nostril).

Post-partum follow-up

Mother-neonate pairs will be reviewed one day, one week and one month post-partum. At each visit, maternal consent for neonatal sampling will be re-affirmed, and respiratory samples will be collected (Table 2). An optional umbilical cord blood sample (Visit 3) and neonatal venous blood sample (Visit 5) will be collected, as well as relevant clinical data (including mode of delivery, infant feeding and use of antimicrobials). Optional breastmilk samples will also be collected at each post-partum visit (up to 5mL each at Visits 3 to 5). Volunteers may decline optional blood and breastmilk sampling without affecting their enrolment in or completion of the rest of the study.

Volunteers' experiences of participation (including acceptability and tolerability of inoculation and maternal and infant sampling) will be captured using optional questionnaires, to help us identify areas for improving volunteer experience in current and future studies.

Optional Visit 6 (15+/-2 weeks post-partum)

At the time of writing, a study amendment is in progress to include an optional study visit at 15+/-2 weeks post-partum (Visit 6). This visit will involve maternal and infant pharyngeal, saliva and venous blood sampling, as well as saliva and oropharyngeal swabs from household contacts under 5 years old. In a recent *N. lactamica* controlled human infection study involving non-pregnant adults, anti-*N. lactamica* immunoglobulin G (IgG) (peak titre and titre increment) correlated inversely with subsequent pharyngeal colonisation density at 28 days post-inoculation (unpublished data), suggesting that IgG may play a role in controlling

For peer review only - http://bmjopen.bmj.com/site/about/guidelines.xhtml
For peer review only

N. lactamica carriage. Total IgG is up to 1.5 times more concentrated in umbilical cord blood compared with maternal blood[23], and the half-life of transplacental maternal IgG is approximately 30 days[24]; thus, infant anti-*N. lactamica* IgG is predicted to be less than 10-20% that of maternal IgG by 15+/-2 weeks (3 to 4 half-lives). Visit 6 therefore offers a valuable opportunity to investigate the association between anti-*N. lactamica* IgG and *N. lactamica* carriage in mother-infant pairs.

Sample processing

N. lactamica identification

Working in a class 2 microbiological safety cabinet in a Containment Level 2 laboratory, all respiratory samples will be suspended in 1mL storage medium (10% glycerol in 0.1% diethylpyrocarbonate [DEPC]-treated water). Agar selective for *N. lactamica* (GC agar supplemented with 5-bromo-4-chloro-3-indolyl-β-galactopyranoside [X-gal]; Thermo Fisher Scientific, Basingstoke, United Kingdom) will be inoculated with sample solution, and all remaining sample solution will be split into 200μL aliquots for storage in a secure, temperature-monitored -80°C freezer. Following 24-48 hours incubation, colonies with typical *N. lactamica* morphology (blue on GC X-gal agar) will undergo identification by oxidase reaction, Gram stain, microscopy, and analytical profile index (API-NH, bioMérieux, Lyon, France) or matrix-assisted laser desorption/ionization time-of-flight (MALDI-TOF; Bruker, Coventry, United Kingdom). *N. lactamica* density will be quantified and isolates will be stored at -80°C. Breastmilk will also be plated onto selective agar to examine for evidence of *N. lactamica*, and residual samples will be stored at -80°C for microbiome analysis.

N. lactamica Y92-1009 strain-specific PCR

We have identified two chromosomal loci present in *N. lactamica* strain Y92-1009 that, when amplified in conventional multiplex PCR and analysed by agarose gel electrophoresis, produce amplicons with a characteristic band pattern[18]. Using the same multiplex PCR on isolates of naturally circulating strains of *N. lactamica*, this band pattern has not been reproduced. A third locus, present in all known strains

For peer review only - http://bmjopen.bmj.com/site/about/guidelines.xhtml

of *N. lactamica*, is included to differentiate between PCR failure (no bands produced) and any non-Y92-1009 strain of *N. lactamica* (band produced, but Y92-1009-specific bands pattern not present). This PCR assay has been validated using genomic DNA and boiled bacterial lysate as a template, with an optimum annealing temperature 65°C and extension time 15 seconds (unpublished data).

Microbiome analysis

Paired mother-infant frozen sample aliquots will be transported to the University of Edinburgh Centre for Inflammation Research for 16S rRNA gene sequencing[6]. Resulting sequence reads will undergo taxonomic classification to compare mothers and infants, and (assuming some neonates become colonised with *N. lactamica*) colonised and uncolonised infants, across different sample niches and study time points.

N. lactamica sequencing

If any neonates become colonised with *N. lactamica*, paired mother-infant *N. lactamica* isolates will undergo whole genome sequencing to assess for differences in *N. lactamica* microevolution in mother-infant pairs. Resulting sequences will be mapped to a closed *N. lactamica* Y92-1009 reference genome for comparison.

Sample size

To our knowledge, this proof-of-concept represents the first respiratory controlled human infection study in pregnancy. As such, it is not possible to accurately define the sample size needed to conclusively rule out induced horizontal transfer of *N. lactamica*. A pragmatic sample size of 20 inoculated volunteers has been selected: based on our earlier work, *N. lactamica* colonisation is expected in 18 (90%) of volunteers inoculated with 10^5 CFU *N. lactamica*. This sample would therefore fail to detect horizontal transfer only if it occurs in less than 1 in 18 infants (<6%), in which case it would be impractical and even unethical to pursue horizontal *N. lactamica* controlled human infection as a method of studying and manipulating infant commensalisation. By one month of

For peer review only - http://bmjopen.bmj.com/site/about/guidelines.xhtml

age, 2.3% of infants naturally carry *N. lactamica*[17]. Based on a plot of lower confidence limits against observed infant *N. lactamica* colonisation for a sample of 18 experimentally-colonised volunteers, infant *N. lactamica* colonisation greater than 22% (95% CI [2.3%, 41.7%]) would demonstrate that inoculation-induced carriage exceeds natural *N. lactamica* carriage (Figure 2).

We expect to screen at least 24 volunteers to reach our target of 20 inoculations, allowing for 10% dropout and 10% baseline natural *N. lactamica* carriage[18]. It is not clear how maternal or neonatal antibiotics may influence *N. lactamica* carriage and microbiota. At the Principal Investigator's discretion, an additional volunteer may be enrolled and inoculated for every volunteer (or neonate) who receives antibiotics after inoculation, up to a maximum of 10 additional volunteers.

Statistical analysis

The primary endpoint will be expressed as a proportion N/M, where:

- M = Number of mothers colonised with *N. lactamica;*
- N = Number of neonates (aged 0-31 days) colonised with the same strain of *N. lactamica* as their own mother;
- "Colonised" = evidence of *N. lactamica* by culture of upper respiratory swabs (using selective media and confirmation by API or MALDI-TOF) on at least one visit;
- "Same strain" = confirmed using Y92-specific *N. lactamica* PCR for inoculated volunteers, or whole genome sequencing for volunteers already naturally carrying *N. lactamica* at screening.

Subgroup analysis will be performed, to compare inoculated mother-infant pairs with naturally colonised mother-infant pairs, and to compare pairs where the mother or infant received antibiotics with pairs not receiving antibiotics.

Specific bioinformatic analysis strategies will depend on the quantity and quality of genomic data produced during the study, and on the rates of *N. lactamica* colonisation observed. Broadly speaking, microbiome bioinformatic pipelines

For peer review only - http://bmjopen.bmj.com/site/about/guidelines.xhtml

(e.g. QIIME2 and R packages) will be used to perform taxonomic classification, calculation of alpha and beta diversity, and data visualisation.

Patient and public involvement

During early study design, an interview-based Patient and Public Involvement (PPI) project was conducted with 12 pregnant women (unpublished data). All 12 women expressed approval that the proposed study should go ahead, 11 of whom would personally have liked to participate. These discussions led to changes in study design (such as offering follow-up visits in the volunteer's own home, and obtaining an umbilical cord rather than an infant venous blood sample at birth). During participant recruitment following study completion, the University of Southampton Public Engagement Research Unit will assist in showcasing the study at public engagement events.

<u>Ethics and dissemination</u>

Ethics and safety

Volunteer safety and ethical study conduct is of paramount importance. This study has been approved by the NHS Research Ethics committee (REC) and the Health Research Authority (HRA), and is sponsored by the University of Southampton. An External Safety Committee has been appointed to provide independent safety oversight. Safety of *N. lactamica* Y92-1009 inoculum is well-characterised: over 400 adults have previously been inoculated with this *N. lactamica* strain (of whom 30 received lyophilised inoculum, and 54 received 10^5 CFU), with no serious adverse reactions reported to date[15] (unpublished data). Due to the use of yeast-based and soya-based products in inoculum production, individuals reporting allergies to yeast or soya will not be eligible for enrolment. Inoculation will take place in a clinical area with access to adult, obstetric and paediatric emergency services. Volunteers will be reviewed by a study doctor immediately prior to and 30 minutes after inoculation.

For peer review only - http://bmjopen.bmj.com/site/about/guidelines.xhtml

Adverse events will be recorded throughout the study, and volunteers will be encouraged to report any concerns (including by telephone to the study doctor out-of-hours). Adverse events will also be recorded for any inoculated withdrawn volunteers, by telephone review at four weeks post-partum. As per the current Good Clinical Practice guideline[25], adverse events are any untoward medical occurrence in a volunteer or their neonate, temporally, but not necessarily causally, related to study participation. Delivery of routine peripartum care is not considered an adverse event. Serious adverse events include hospitalisation (or prolongation of hospitalisation for routine peripartum care), persistent or significant disability, a life-threatening occurrence, or death from any cause.

Mitigation strategies are in place to minimise the risk of SARS-CoV2 transmission to participants and study staff. Where possible, study visits will be conducted in the volunteer's home, practicing social distancing and appropriate personal protective equipment for non-aerosol generating procedures. If participants report symptoms of or contact with anyone with suspected or confirmed SARS-CoV2 infection, study visits will be rescheduled until testing or self-isolation as necessary.

Data management and dissemination

All data will be stored in link-anonymised format using paper case report forms stored securely at the research site. Fully anonymised sequencing data will be uploaded to a curated online data repository. Research findings will be published in peer-reviewed journals as soon as is practicable, with an online link to the final approved study protocol. Participants will be provided with a lay summary of the study results once available.

Potential benefits and study impact

To our knowledge, this is the first respiratory controlled human infection study in pregnancy, and, if successful, would be the first to demonstrate induced person-to-person commensal transmission. This novel model could be used to

For peer review only - http://bmjopen.bmj.com/site/about/guidelines.xhtml

characterise microbiome and immunological changes associated with induced commensalisation. Inducing *N. lactamica* colonisation in neonates may also provide a new strategy for reducing *N. meningitidis* colonisation and even disease in infants. Although many neonates are protected by trans-placental maternal antibodies, invasive meningococcal disease does rarely occur in infants, especially before vaccination[26]. Looking ahead, improved understanding of microbiome evolution following controlled human infection is necessary before inoculation in neonates (rather than in pregnant adults) can be considered.

Our team have recently trialled nasal inoculation in healthy non-pregnant adults using genetically-modified (GM) *N. lactamica* that expresses the meningococcal antigen *Neisseria* adhesin A (NadA)[27]. This GM inoculum has proved safe and effective at eliciting immune responses to NadA, providing a proof-of-concept that a harmless commensal may be used as a vehicle for prolonged mucosal exposure to an antigen of interest. Looking ahead, GM *N. lactamica* expressing antigens from other clinically-important paediatric respiratory pathogens could be explored, to complement existing childhood vaccination.

For peer review only

Tables

**Primary endpoint**	**Secondary endpoints**
1. Confirmation of neonatal (0-31 days) *N. lactamica* colonisation by selective culture of nasopharyngeal and saliva samples.	1. Confirmation of neonatal *N. lactamica* Y92-1009 colonisation by strain-specific PCR.<br>2. *N. lactamica* colonisation density in inoculated volunteers compared with their infants across study time points and sample niche.<br>3. Microbiome composition across study time points and sample niche in inoculated volunteers compared with their infants, and in colonised compared with uncolonised infants.<br>4. *N. lactamica* genome sequence for isolates derived from inoculated volunteers compared with their infants across study time points and sample niche.
**Safety endpoints**	
1. Percentage of inoculated volunteers with adverse reactions or serious adverse events within the study period.<br>2. Percentage of neonates with serious adverse events within the study period.	

Table 1. Primary, secondary and safety study endpoints. PCR: polymerase chain reaction.

	Screening (Visit 1)	Inoculation (Visit 2)	Birth (Visit 3)	Follow-up (Visits 4 and 5)		Optional (Visit 6)
**Timeline**	**34+0 to 36+6 weeks gestation**	**36+0 to 37+6 weeks gestation**	**0-24 hours post-partum**	**7 +/- 3 days post-partum**	**28 +/- 3 days post-partum**	**15 +/- 2 weeks post-partum**
**Participant Information Sheet and consent form**	+					
**Clinical review**	+	+	+	+	+	+
***N. lactamica* nasal inoculation**		+ (Unless already colonised)				
**Nasopharyngeal swabs (mother)**	+	+	+	+	+	+
**Oropharyngeal swabs (mother)**	+	+	+	+	+	+
**Saliva swabs (mother)**	+	+	+	+	+	+
**Nasopharyngeal swabs (infant)**			+	+	+	+

For peer review only - http://bmjopen.bmj.com/site/about/guidelines.xhtml

**Saliva swabs (infant)**			+	+	+	+
**Umbilical cord blood**			Optional			
**Infant venous blood (2ml)**					Optional	Optional
**Maternal breast milk**			Optional	Optional	Optional	Optional
**Maternal venous blood (5ml)**						Optional
**Oropharyngeal & saliva swabs (under 5 years)**						Optional

Table 2: Study visits and activities.

Inclusion criteria (all must apply):

- Healthy adult aged 18 years or over on the day of enrolment.
- Singleton pregnancy, 34+0 to 36+6 weeks gestation on the day of enrolment.
- Documentation of a 20-week ultrasound scan with no life-limiting congenital anomalies, and no maxillofacial, otorhinolaryngolocial or neuroanatomical anomalies.
- Able and willing (in the Investigator's opinion) to comply with all study requirements.
- Able and willing to give written informed consent to participate in the study.
- Booked to receive antenatal care at University Hospital Southampton NHS Foundation Trust (including any associated community antenatal care facilities), United Kingdom.

Exclusion criteria (none must apply):

- Any confirmed or suspected immunosuppressive or immunocompromised state, including: HIV infection; asplenia; recurrent severe infections; or use of immunosuppressant medication (for more than 14 days within the past 6 months, excluding topical and inhaled steroids).
- Planned use of immunosuppressant medication in later pregnancy or post-partum.

For peer review only

For peer review only - http://bmjopen.bmj.com/site/about/guidelines.xhtml

- Occupational, household or intimate contact with any immunosuppressed persons.
- Participation within the last 12 weeks in a clinical trial involving receipt of an investigational product, or planned use of an investigational product during the study period.
- Prior participation at any time in research studies involving inoculation with *N. lactamica.*
- Use of oral or intravenous antibiotics within 30 days prior to the inoculation visit.
- Planned use of oral or intravenous antibiotics at any time during the study period (e.g. for planned elective caesarean section or group B streptococcus colonisation).
- Allergy to soya or yeast.
- Previous stillbirth or neonatal death.
- Pre-pregnancy diabetes mellitus.
- Any other finding that may (in the Investigator's opinion): increase the risk to the volunteer (or their fetus/infant or close contacts) of participating in the study; affect the volunteer's ability to participate in the study and complete follow-up; or impair interpretation of study data.

467 Table 3. Inclusion and exclusion criteria. NHS: National Health Service; HIV:
468 human immunodeficiency virus.

For peer review only - http://bmjopen.bmj.com/site/about/guidelines.xhtml

References

1. Roser M et al. Child and Infant Mortality. OurWorldInData.org Nov 2019. Retrieved Dec 2020 from: https://ourworldindata.org/child-mortality.
2. Gostin L et al. The public health crisis of underimmunisation: a global plan of action. Lancet Infect Dis 2020;20(1):e11-16.
3. Laxminarayan R et al. Access to effective antimicrobials: a worldwide challenge. Lancet 2016;387(10014):168-75.
4. Theodosiou AA et al. The infant pharyngeal microbiomes: origin, impact and manipulation. Curr Opin Infect Dis 2020;33(6):548-555.
5. Teo S et al. Dynamics of airway microbiota identify a critical window for interplay of pathogenic bacteria and allergic sensitization in childhood respiratory disease. Cell Host Microbe 2018;24(3):341–352.
6. Luna P et al. The association between anterior nares and nasopharyngeal microbiota in infants hospitalized for bronchiolitis. Microbiome 2018; 6(1):2.
7. Bosch A et al. Maturation of the infant respiratory microbiota, environmental drivers, and health consequences.Am J Respir Crit Care Med 2017;196(12):1582–1590.
8. Thorsen J et al. Infant airway microbiota and topical immune perturbations in the origins of childhood asthma. Nat Commun 2019;10(1):5001.
9. Man W et al. Loss of microbial topography between oral and nasopharyngeal microbiota and development of respiratory infections early in life. Am J Respir Crit Care Med 2019;200(6):760–770.
10. de Steenhuijsen Piters W et al. The role of the local microbial ecosystem in respiratory health and disease. Philos Trans R Soc B Biol Sci 2015;370(1675):20140294.
11. Ferretti P et al. Mother-to-infant microbial transmission from different body sites shapes the developing infant gut microbiome. Cell Host Microbe 2018;24(1):133–145.
12. Theodosiou AA et al. Manipulating the infant respiratory microbiomes to improve clinical outcomes: A review of the literature. J Infect 2021;82(6):247-252.
13. Shinefield H et al. Bacterial interference: its effect on nursery-acquired infection with *Staphylococcus aureus*. V. An analysis and interpretation. Am J Dis Child 1963;105:683–8.
14. Sprunt K et al. Abnormal colonization of neonates in an ICU: conversion to normal colonization by pharyngeal implantation of alpha haemolytic *Streptococcus* strain 215. Pediatr Res 1980;14(4):308–313.
15. Hao Q et al. Probiotics for preventing acute upper respiratory tract infections. Cochrane Database Syst Rev 2015;(2):CD006895.
16. Elias J et al. Are probiotics safe for use during pregnancy & lactation? Can Fam Physician 2011;57(3):299-301.
17. Deasy A et al. Nasal Inoculation of the Commensal *Neisseria lactamica* Inhibits Carriage of *Neisseria meningitidis* by Young Adults. Clin Infect Dis 2015;60(10):1512-20.
18. Dale AP et al. *Neisseria lactamica* Controlled Human Infection Model. Methods in Molecular Biology (Clifton, N.J.) 2022 ;2414:387-404.
19. Pandey A et al. Microevolution of *Neisseria lactamica* during nasopharyngeal colonisation induced by controlled human infection. Nat Commun 2018;9(1):4753.
20. Bennett J et al. Genetic diversity and carriage dynamics of *Neisseria lactamica* in infants. Infect Immun 2005;73(4):2424.

For peer review only - http://bmjopen.bmj.com/site/about/guidelines.xhtml

21. Olsen S et al. Pharyngeal carriage of *N meningitidis* and *N lactamica* in households with infants within areas with high and low incidences of meningococcal disease. Epidemiol Infect 1991;106(3):445-57.
22. Gold R et al. Carriage of *N meningitidis* and *N lactamica* in infants and children. J Infect Dis 1978;137(2):112.
23. Clements T et al. Update on Transplacental Transfer of IgG Subclasses: Impact of Maternal and Fetal Factors. Front Immunol 2020;11:1920.
24. Oguti B et al. The half-life of maternal transplacental antibodies against diphtheria, tetanus, and pertussis in infants: an individual participant data meta-analysis. Vaccine 2022;40(3):450-458.
25. International Conference on Harmonisation E6 Good Clinical Practice CPMP/ICH/135/95. Retrieved Feb 2022 from: https://www.ich.org/page/efficacy-guidelines#6-2.
26. Kiray Bas et al. Neonatal infection with *Neisseria meningitidis*: analysis of a 97-year period plus case study. J Clin Microbiol 2014;52(9):3478-82.
27. Laver JR et al. A recombinant commensal bacteria elicits heterologous antigen-specific immune responses during pharyngeal carriage. Sci Transl Med. 2021;13(601).

Contributorship statement

A Theodosiou prepared the manuscript, led the study design, and is the Principal Investigator and overall guarantor for the study. C Jones and R Read are the Chief Investigators and provided clinical and academic supervision. A Dale was involved in clinical and microbiological study design, D Cleary supervised genomic and bioinformatic study design, and J Laver supervised laboratory and molecular study design. All authors reviewed, edited and approved the final manuscript.

Funding statement

This work is supported by a Medical Research Council Clinical Research Training Fellowship (grant number MR/V002015/1) awarded to Dr Anastasia A Theodosiou. Robert C Read is an NIHR Senior Investigator. The funders have not had input into the design or execution of the study or the preparation of this manuscript.

Competing interests statement

The authors have no competing interests to declare.

For peer review only - http://bmjopen.bmj.com/site/about/guidelines.xhtml

Additional trial registration and SPIRIT checklist data

Primary trial registration number (ClinicalTrials.gov): NCT04784845 (registered 5th March 2021)

Secondary identifying numbers: IRAS (NHS REC) 290865, ERGO (Sponsor) 61640

Date of first enrolment: 25th October 2021

Recruitment status: recruiting

Research site: University Hospital Southampton NHS Foundation Trust, Tremona Road, Southampton SO166YD, United Kingdom

Current protocol version: Version 1.3 (22nd June 2021). At the time of writing, Protocol Version 1.4 (4th February 2022) is being reviewed by the Research Ethics Committee as part of a substantial study amendment.

Contact for public and scientific queries: Dr A Theodosiou (PI) at1u17@soton.ac.uk

Contact for study Sponsor: University of Southampton, rgoinfo@soton.ac.uk

Sponsor insurance statement: A specialist insurance policy is in place, which would operate in the event of any volunteer suffering harm as a result of involvement in the study.

Data monitoring: To be overseen by the Sponsor. For a small pilot study investigating basic science rather than clinical outcomes, a formal external Data Monitoring Committee was not felt to be appropriate by the Sponsor or the Research Ethics Committee.

For peer review only

Figure legends

Figure 1: Study overview flowchart. Nlac: *Neisseria lactamica*; m: months; w: weeks; h: hours; API: analytical profile index; MALDI [MALDI-TOF]: matrix-assisted laser desorption/ionization time-of-flight; CFU: colony forming units; PCR: polymerase chain reaction; DNA: deoxyribonucleic acid

Figure 2: Lower confidence limit plot for inoculation-induced *N. lactamica* (Nlac) colonisation.

For peer review only

For peer review only - http://bmjopen.bmj.com/site/about/guidelines.xhtml

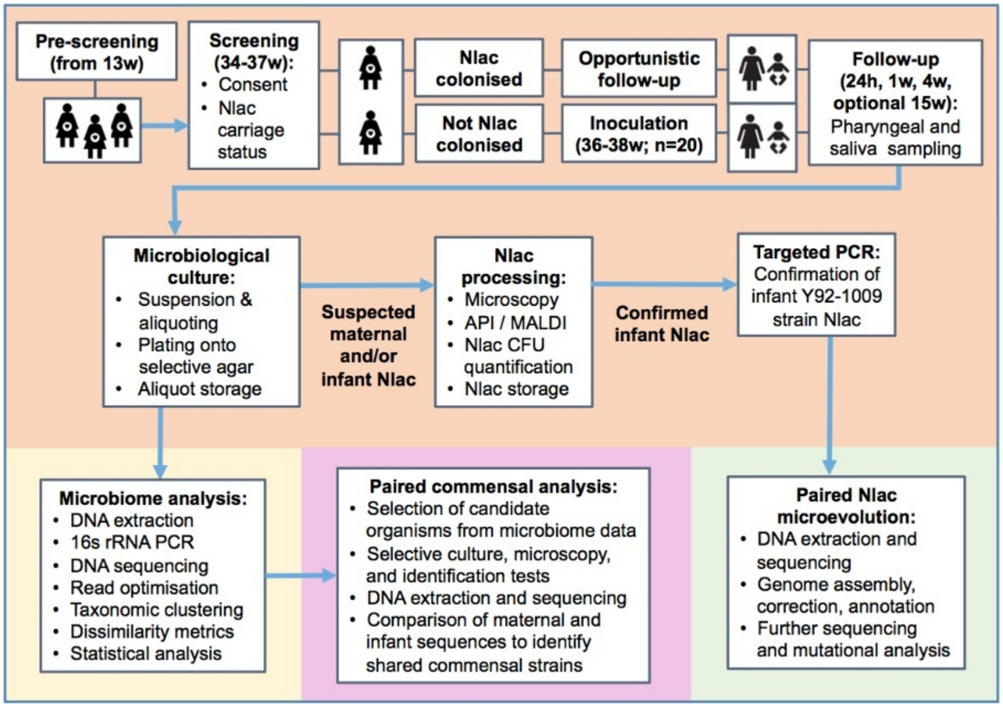

Figure 1: Study overview flowchart. Nlac: Neisseria lactamica; m: months; w: weeks; h: hours; API: analytical profile index; MALDI [MALDI-TOF]: matrix-assisted laser desorption/ionization time-of-flight; CFU: colony forming units; PCR: polymerase chain reaction; DNA: deoxyribonucleic acid

159x111mm (144 x 144 DPI)

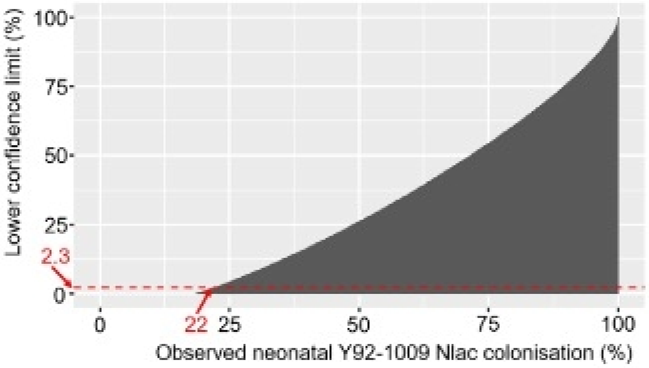

Figure 2: Lower confidence limit plot for inoculation-induced N. lactamica (Nlac) colonisation.

116x66mm (72 x 72 DPI)

SPIRIT 2013 Checklist: Recommended items to address in a clinical trial protocol and related documents*

Section/ Item	Item No	Description	Page/ Line
**Administrative information**			
Title	1	Descriptive title identifying the study design, population, interventions, and, if applicable, trial acronym	1/1
Trial registration	2a	Trial identifier and registry name. If not yet registered, name of intended registry	22/559
	2b	All items from the World Health Organization Trial Registration Data Set	22/557
Protocol version	3	Date and version identifier	22/565
Funding	4	Sources and types of financial, material, and other support	21/545
Roles and responsibilities	5a	Names, affiliations, and roles of protocol contributors	1/9
	5b	Name and contact information for the trial sponsor	22/570
	5c	Role of study sponsor and funders, if any, in study design; collection, management, analysis, and interpretation of data; writing of the report; and the decision to submit the report for publication, including whether they will have ultimate authority over any of these activities	21/549
	5d	Composition, roles, and responsibilities of the coordinating centre, steering committee, endpoint adjudication committee, data management team, and other individuals or groups overseeing the trial, if applicable (see Item 21a for data monitoring committee)	N/A
**Introduction**			
Background and rationale	6a	Description of research question and justification for undertaking the trial, including summary of relevant studies (published and unpublished) examining benefits and harms for each intervention	7/169
	6b	Explanation for choice of comparators	17/462
Objectives	7	Specific objectives or hypotheses	8/197

For peer review only

Trial design	8	Description of trial design including type of trial (eg, parallel group, crossover, factorial, single group), allocation ratio, and framework (eg, superiority, equivalence, exploratory)	8/182
**Methods: Participants, interventions, and outcomes**			
Study setting	9	Description of study settings (eg, community clinic, academic hospital) and list of countries where data will be collected. Reference to where list of study sites can be obtained	18/467
Eligibility criteria	10	Inclusion and exclusion criteria for participants. If applicable, eligibility criteria for study centres and individuals who will perform the interventions (eg, surgeons, psychotherapists)	18/467
Interventions	11a	Interventions for each group with sufficient detail to allow replication, including how and when they will be administered	8/209
	11b	Criteria for discontinuing or modifying allocated interventions for a given trial participant (eg, drug dose change in response to harms, participant request, or improving/worsening disease)	N/A
	11c	Strategies to improve adherence to intervention protocols, and any procedures for monitoring adherence (eg, drug tablet return, laboratory tests)	N/A
	11d	Relevant concomitant care and interventions that are permitted or prohibited during the trial	N/A
Outcomes	12	Primary, secondary, and other outcomes, including the specific measurement variable (eg, systolic blood pressure), analysis metric (eg, change from baseline, final value, time to event), method of aggregation (eg, median, proportion), and time point for each outcome. Explanation of the clinical relevance of chosen efficacy and harm outcomes is strongly recommended	17/462
Participant timeline	13	Time schedule of enrolment, interventions (including any run-ins and washouts), assessments, and visits for participants. A schematic diagram is highly recommended (see Figure)	Figure 1
Sample size	14	Estimated number of participants needed to achieve study objectives and how it was determined, including clinical and statistical assumptions supporting any sample size calculations	12/332
Recruitment	15	Strategies for achieving adequate participant enrolment to reach target sample size	9/226

For peer review only - http://bmjopen.bmj.com/site/about/guidelines.xhtml

**Methods: Data collection, management, and analysis**			
Data collection methods	18a	Plans for assessment and collection of outcome, baseline, and other trial data, including any related processes to promote data quality (eg, duplicate measurements, training of assessors) and a description of study instruments (eg, questionnaires, laboratory tests) along with their reliability and validity, if known. Reference to where data collection forms can be found, if not in the protocol	9/235
	18b	Plans to promote participant retention and complete follow-up, including list of any outcome data to be collected for participants who discontinue or deviate from intervention protocols	15/411
Data management	19	Plans for data entry, coding, security, and storage, including any related processes to promote data quality (eg, double data entry; range checks for data values). Reference to where details of data management procedures can be found, if not in the protocol	15/428
Statistical methods	20a	Statistical methods for analysing primary and secondary outcomes. Reference to where other details of the statistical analysis plan can be found, if not in the protocol	13/359
	20b	Methods for any additional analyses (eg, subgroup and adjusted analyses)	13/369
	20c	Definition of analysis population relating to protocol non-adherence (eg, as randomised analysis), and any statistical methods to handle missing data (eg, multiple imputation)	N/A
**Methods: Monitoring**			
Data monitoring	21a	Composition of data monitoring committee (DMC); summary of its role and reporting structure; statement of whether it is independent from the sponsor and competing interests; and reference to where further details about its charter can be found, if not in the protocol. Alternatively, an explanation of why a DMC is not needed	22/575
	21b	Description of any interim analyses and stopping guidelines, including who will have access to these interim results and make the final decision to terminate the trial	N/A
Harms	22	Plans for collecting, assessing, reporting, and managing solicited and spontaneously reported adverse events and other unintended effects of trial interventions or trial conduct	15/409

For peer review only - http://bmjopen.bmj.com/site/about/guidelines.xhtml

Auditing	23	Frequency and procedures for auditing trial conduct, if any, and whether the process will be independent from investigators and the sponsor	N/A
**Ethics and dissemination**			
Research ethics approval	24	Plans for seeking research ethics committee/institutional review board (REC/IRB) approval	2/43
Protocol amendments	25	Plans for communicating important protocol modifications (eg, changes to eligibility criteria, outcomes, analyses) to relevant parties (eg, investigators, REC/IRBs, trial participants, trial registries, journals, regulators)	10/270
Consent or assent	26a	Who will obtain informed consent or assent from potential trial participants or authorised surrogates, and how (see Item 32)	9/237
	26b	Additional consent provisions for collection and use of participant data and biological specimens in ancillary studies, if applicable	N/A
Confidentiality	27	How personal information about potential and enrolled participants will be collected, shared, and maintained in order to protect confidentiality before, during, and after the trial	15/428
Declaration of interests	28	Financial and other competing interests for principal investigators for the overall trial and each study site	21/553
Access to data	29	Statement of who will have access to the final trial dataset, and disclosure of contractual agreements that limit such access for investigators	15/428
Ancillary and post-trial care	30	Provisions, if any, for ancillary and post-trial care, and for compensation to those who suffer harm from trial participation	22/572
Dissemination policy	31a	Plans for investigators and sponsor to communicate trial results to participants, healthcare professionals, the public, and other relevant groups (eg, via publication, reporting in results databases, or other data sharing arrangements), including any publication restrictions	15/428
	31b	Authorship eligibility guidelines and any intended use of professional writers	21/539
	31c	Plans, if any, for granting public access to the full protocol, participant-level dataset, and statistical code	15/428

*It is strongly recommended that this checklist be read in conjunction with the SPIRIT 2013 Explanation & Elaboration for important clarification on the items. Amendments to the protocol should be tracked and dated. The SPIRIT checklist is copyrighted by the SPIRIT Group under the Creative Commons "Attribution-NonCommercial-NoDerivs 3.0 Unported" license.

For peer review only
